# Binaphthyl-anchored antibacterial tripeptide derivatives with hydrophobic C-terminal amino acid variations

**DOI:** 10.3762/bjoc.8.142

**Published:** 2012-08-09

**Authors:** John B Bremner, Paul A Keller, Stephen G Pyne, Mark J Robertson, K Sakthivel, Kittiya Somphol, Dean Baylis, Jonathan A Coates, John Deadman, Dharshini Jeevarajah, David I Rhodes

**Affiliations:** 1 School of Chemistry, University of Wollongong, Wollongong, NSW 2522, Australia; 2 Avexa Ltd, 576 Swan St, Richmond, Vic 3121, Australia; 3 Chemocopeia Pty Ltd, 114 Kay St, Carlton, Melbourne, Vic 3053, Australia; 4 JDJ Bioservices Pty Ltd, 576 Swan St, Richmond, Vic 3121, Australia

**Keywords:** antibacterials, binaphthyls, cationic peptides, peptides, resistance, VISA, VRE

## Abstract

The facile synthesis of seven new dicationic tripeptide benzyl ester derivatives, with hydrophobic group variations in the C-terminal amino acid component, is described. Moderate to good activity was seen against Gram-positive bacteria in vitro. One cyclohexyl-substituted compound **2c** was tested more widely and showed good potency (MIC values ranging from 2–4 μg/mL) against antibiotic-resistant strains of *Staphylococcus aureus* and Enterococci (VRE, VSE), and against *Staphylococcus epidermidis*.

## Introduction

**Figure 1:**
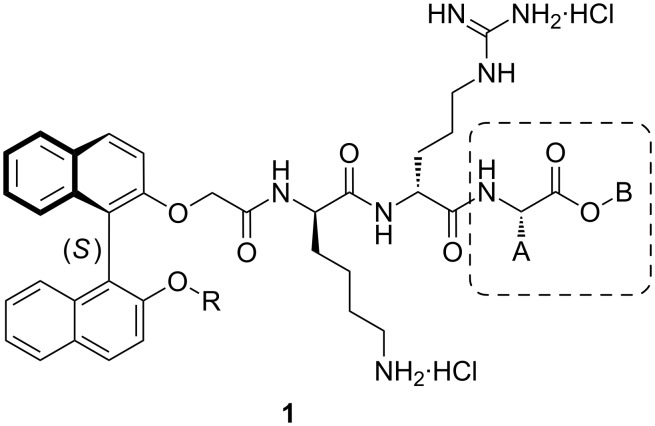
Binaphthyl-anchored tripeptide derivatives **1**.

Among the most pressing challenges in current healthcare is the resistance of bacterial human pathogenic organisms to antibiotics [1–2], and of particular concern is the resistance to the cationic glycopeptide, vancomycin [3–4]. This challenge is being addressed in a number of ways, which include both detailed studies aimed at the further understanding of the mechanism of this resistance, as well as the development of new large glycopeptide analogues containing amine sites that can be protonated, such as telavancin, oritavancin and dalbavancin [5]. An alternative approach to meeting this resistance challenge, at least in part, is through the design and synthesis of smaller cationic peptidic compounds incorporating features that could circumvent the vancomycin resistance mechanism. In much of our work we have been concerned with binaphthyl-based dicationic peptide derivatives, for example the acyclic tripeptides of type **1** (Figure 1; for example A = CH_2_CH=CH_2_, B = CH_2_Ph, R = CH_2_CH_2_CH(CH_3_)_2_, MIC against *S. aureus* 4 μg/mL [6]), which show significant promise as antibacterials [6–8]. In the development of these antibacterials, it became apparent that the nature of the C-terminal amino acid derivative was significant, with indications that the hydrophobic groups at the terminus (Figure 1, group B) [6] and at the α-carbon of the amino acid moiety (Figure 1, group A) [6–7] were important. In further exploration of the structural space in the latter area, while maintaining benzyl ester functionality at the terminus itself (a free carboxylic acid unit at the C-terminal was deleterious to activity [8]), we envisaged the introduction of two alkyl substituent units incorporated in a ring system at this carbon, resulting in the target compounds **2a**–**d**. Two further compounds based on a β–alanine unit with disubstitution at the α- or β-carbons of this unit, **2f** and **2e** respectively, were accessed with a view to assessing the effect of variation in the spatial disposition of the cycloalkyl or oxacycloalkyl ring on antibacterial activity. The conformationally less restricted *gem* diethyl-substituted compound **2g** was also targeted in order to make antibacterial activity comparisons with the five-membered ring analogue **2b**. The results of these synthetic and antibacterial testing studies are reported in this paper.

## Results and Discussion

**Scheme 1:**
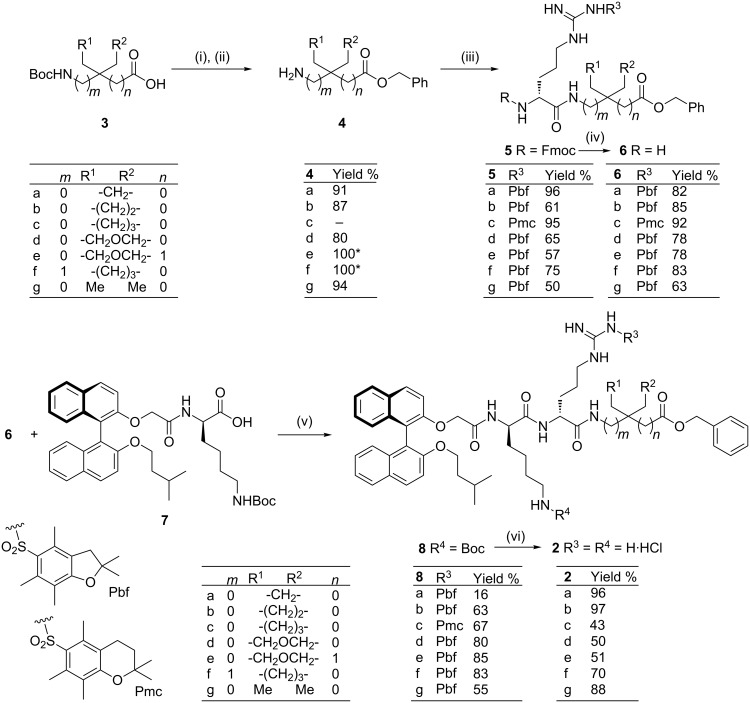
Synthesis of compounds **2a**–**g**. Reagents and conditions: (i) **2a**–**b**,**d**–**g**, BnBr, acetone or THF, K_2_CO_3_, reflux, 3–12 h; (ii) TFA, DCM, rt, 1–2 h, 2 M HCl/Et_2_O (HCl salts); (iii) **5a**–**f**: (DIPEA), Fmoc-(*R*)-Arg(Pmc)-OH (**5c**) or Fmoc-(*R*)-Arg(Pbf)-OH (**5a**–**b**, **d**–**f**), EDCI, HOBt, CH_3_CN, rt, 3–16 h; **5g**: DIPEA, Fmoc-(*R*)-Arg(Pbf)-OH, BOP, CH_3_CN, rt, 3–16 h (iv) piperidine, CH_3_CN, rt, 3–12 h; (v) EDCI, HOBt, CH_3_CN, rt, 3–72 h; (vi) TFA/CH_2_Cl_2_ (1:1), rt, 2–16 h, then 2 M HCl/Et_2_O. *Isolated as the HCl salt.

A concise and flexible approach to all the target compounds **2a**–**g** was used, starting from the commercially available amino acid derivatives **3** and proceeding via the amino benzyl esters **4** (Scheme 1), with the exception of the synthesis of **2c** in which the commercially available **4c** was used as the starting material. Addition of the protected central arginine unit by diimide- or BOP-induced amide bond formation, followed by selective Fmoc removal from the respective intermediates **5**, then provided access to the key intermediate amines **6a**–**g** (Scheme 1). Diimide-mediated coupling of the previously reported lysine containing (*S*)-binaphthyl acid derivative **7** [8] then afforded the protected tripeptides **8a**–**g**. Removal of the Pmc (or Pbf) and Boc protecting groups in one pot was then achieved by exposure to trifluoroacetic acid, followed by trifluoroacetate/chloride ion exchange on treatment with an excess of HCl in diethyl ether, and finally evaporation to afford the salts **2a**–**g** in good overall yields.

The structures of the final compounds were supported by ^1^H and ^13^C NMR spectroscopy and high-resolution mass spectrometry. Details of the synthesis and spectral data are given in the experimental section for the representative compound **2c**. The experimental and spectroscopic data for all the other compounds are included in Supporting Information File 1.

The peptidic dihydrochloride salts **2a**–**g** were tested against the Gram-positive bacteria *Staphylococcus aureus* (ATCC 6538) and four clinical isolates of vancomycin-resistant (and sensitive) enterococci (VRE; *Enterococcus faecium*), and the results are shown in Table 1; some compounds were also tested against *Staphylococcus epidermidis* (ATCC 12228). Vancomycin was used as a positive control, and showed a rounded MIC (minimum inhibitory concentration) value of 2–3 μg/mL against *S. aureus* and MIC values of 2, >25, >25 and 3 μg/mL against the vancomycin sensitive and partially resistant enterococci strains, VRE_243_, VRE_449_, VRE_820_ and VRE_987_, respectively (Table 1). For comparison purposes, data for the previously reported [6] isobutyl-substituted analogue **9** (Table 2) are also included.

**Table 1:** MIC values (μg/mL) against *S. aureus*, *S. epidermidis* and Enterococci of the tripeptide benzyl ester derivatives **2a**–**g** and **9** [6] as their dihydrochloride salts.^a^

Compound	*S. aureus*	*S. epidermidis*	VRE_243_	VRE_449_	VRE_820_	VRE_987_

**2a**	4	–	62	31	31	62
**2b**	3	–	62	31	31	62
**2c**	2	–	31	16	16	31
**2d**	3	3	>25	>25	>25	>25
**2e**	3	3	>25	>25	>25	>25
**2f**	3–6	3	25	25	12	25
**2g**	2	2	16	16	16	31
**9**	4	–	16	16	8	16
Vancomycin	2–3	3	2	>25	>25	3

^a^All MIC values have been rounded to whole numbers.

While MIC micromolar values should be used for comparative analysis of activities, the more commonly used concentration of micrograms per milliliter is retained in this case. The micromolar values are barely different from the latter, as the molecular weights for all of the compounds are similar (959–1001; vancomycin·HCl, 1486). In most cases, incorporation of a hydrophobic alkyl ring in the chain adjacent to the ester functionality results in good activity against *S. aureus* and *S. epidermidis*, but a greater variation of activity was seen with the enterococcal strains. The systems with four- and five-membered cycloalkyl rings (**2a** and **2b**) displayed similar activities, while the cyclohexyl analogue **2c** was somewhat better. Reducing the hydrophobicity while increasing the hydrophilicity of the six-membered ring in **2c** through inclusion of a ring oxygen atom, as in **2d**, had a significant detrimental effect on the activity against the vancomycin-resistant strains VRE_449_ and VRE_820_. Interestingly, the placement of an extra methylene group in the chain, either on the carboxylic ester side (**2e**) or the amino side (**2f**), had no or little effect on antibacterial activity, regardless of whether a ring oxygen atom was present or not. It appeared that moving the hydrophobic ring substituent position by a small amount in the terminal amino acid unit could be tolerated in these systems.

The diethyl-substituted peptide derivative **2g** was more active than the constrained ring comparator compound **2b** against the enterococci, including the vancomycin resistant isolates VRE_449_ and VRE_820_, but both had similarly good potencies against *S. aureus*. In contrast, compound **2c** with the cyclohexyl ring was somewhat more active than the isobutyl-substituted analogue **9**. It would thus appear that greater coverage of the hydrophobic space available in this region is required for good to moderate activity against both the staphylococci and enterococci.

As a result of its promising initial activity profile, the tripeptide derivative **2c** was subjected to further evaluation (Table 2). It was tested against methicillin-sensitive (MSSA), methicillin-resistant (MRSA) and vancomycin-intermediate (VISA) *S. aureus*, *S. epidermis* and vancomycin-resistant (VRE) and vancomycin-sensitive (VSE) enterococci. The test compound **2c** showed good activity against all these Gram-positive bacteria with MIC values in the range of 2–4 µg/mL. Again, for comparison purposes, results for the published analogue **9** [6], containing a single isobutyl group in place of the cyclohexyl group, are also included in Table 2. This tripeptide derivative **9** showed similar, if slightly weaker, antibacterial activity against these strains, apart from VSE, against which it was significantly less potent than **2c**.

**Table 2:** MIC values of compounds **2c** and **9** against Gram-positive isolates.

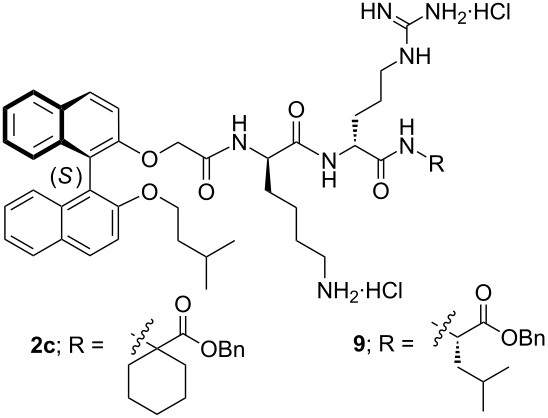			

Strain^a^	MIC_50_ (range) or MIC [μg/mL]^b^		

	**2c**	**9** [6]	Vancomycin

*S. aureus*			
MSSA (8)	2–4	6	1–2
MRSA (7)	2–4	6	1–2
VISA (1)	4	6	6–8
*S. epidermidis* (3)	2	3	2–3

*E. faecium*			
VRE (4)	4	2	>32
VSE (4)	2–4	16	2–8

^a^Compound **2c** was tested against a variety of strains (number in brackets) of *S. aureus*, *S. epidermidis* and *E. faecium*, while compound **9** was tested against only one strain of each organism. Where the MIC was the same for each strain, no range is given. ^b^Where only one strain was tested, the value given is a MIC. *S. epidermidis* = *Staphylococcus epidermidis*; *E. faecium* = *Enterococcus faecium*; MSSA = methicillin-sensitive *S. aureus*; MRSA = methicillin-resistant *S. aureus*; VISA = vancomycin-intermediate *S. aureus*; VRE = vancomycin-resistant enterococci; VSE = vancomycin-sensitive enterococci. MIC = minimum inhibitory concentration [μg/mL; rounded to whole numbers].

Although the mode of action of the tripeptide derivatives has not been established, our earlier results on compounds of type **1** (Figure 1) and related cyclic systems implicated the possibility of more than one mode of action [8]. As noted for other cationic peptide derivatives [9–12], cell membrane damage could well be one of these actions, together with some more specific interactions. Dual-action type behaviour has been shown for the vancomycin analogue telavancin, which affects cell wall synthesis and the integrity of the cell membrane [13].

## Conclusion

In conclusion, seven novel binaphthyl-anchored tripeptide derivatives have been prepared and tested for antibacterial activity against *S. aureus* and four clinical enterococcal strains. The dicationic derivatives **2c** and **2g** showed the best overall activities. In addition, compound **2c** with a hydrophobic cyclohexyl substituent originating from the α-position of the C-terminal amino acid ester, showed good activity against *S. epidermidis* and MSSA, MRSA, VISA and VRE organisms. Our results confirmed the positive contribution to good Gram-positive antibacterial activity, engendered by filling of a hydrophobic area close to the C-terminus of these acyclic tripeptide derivatives.

## Experimental

General notes were those detailed previously in the supporting information for reference [8].

**Benzyl 1-((*R*)-3-amino-1-aza-6-(3,4-dihydro-2,2,5,7,8-pentamethyl-2*H*-1-benzopyran-6-yl)sulfonyl]guanidino)-2-oxohexan)cyclohexanecarboxylate (6c).** This compound was prepared in two steps. To a solution of the amine **4c** [14] (115 mg, 0.49 mmol) in CH_3_CN (5 mL) at rt was added HOBt (1.2 equiv), EDCI (1.2 equiv) and the acid Fmoc-(*R*)-Arg(Pmc)-OH [8] (318 mg, 0.48 mmol). The mixture was stirred for ca. 3 h, then the solvent was removed under reduced pressure, and the resulting residue was subjected to silica gel column chromatography (MeOH/CH_2_Cl_2_, 1:99–4:96 as the eluent) to afford the Fmoc-protected precursor **5c** as a white foamy solid (402 mg, ESIMS *m*/*z*: [M + H]^+^ 877.9 (100%)). This precursor **5c** (200 mg, 0.238 mmol) was then directly deprotected by being stirred in 1 equiv of piperidine/acetonitrile (5 mL per 0.1 mmol of substrate) for 12 h at rt. The solvent was removed under reduced pressure, and the crude product was purified by flash column chromatography (silica gel; CH_2_Cl_2_/MeOH 15:1) to afford the desired amine **6c** as a white solid (141 mg, 87% two steps). ^1^H NMR (500 MHz, CDCl_3_) δ 1.19–1.44 (m, 16H), 1.30 (s, 6H, 2CH_3_ (Pmc)), 2.34 (br s, NH_2_), 2.10 (s, 3H, CH_3_ (Pmc)), 2.57 (s, 3H, CH_3_ (Pmc)), 2.58 (s, 3H, CH_3_ (Pmc)), 2.61 (t, *J* = 6.5 Hz, 2H, CH_2_ (Pmc)), 3.05–3.29 (m, 2H, CH_2_N), 3.39–3.50 (m, 1H, CH (Arg)), 5.06 (ABq, *J =* 12.6 Hz, 1H), 5.09 (ABq, *J =* 12.6 Hz, 1H), 6.41 (s, NH), 7.26–7.41 (m, 5H, ArH), 7.80 (s, NH); ^13^C NMR (125 MHz, CDCl_3_) δ 12.0, 17.4, 18.4, 21.3, 24.5, 25.0, 25.3, 26.7, 26.75, 31.6, 31.9, 32.4, 32.7, 40.5, 54.1, 57.8, 58.4, 66.7, 73.5, 117.8, 123.9, 127.9, 128.1, 128.4, 133.3, 134.6, 135.3, 135.8, 153.4, 156.3, 174.0, 174.4; ESIMS *m*/*z*: [M + H]^+^ 656.3 (100%).

**Benzyl 1-((3*R*,6*R*)-3-(3-[(3,4-dihydro-2,2,5,7,8-pentamethyl-2*H*-1-benzopyran-6-yl)sulfonyl]guanidinopropyl)-9-((*S*)-2'-(3-methylbutoxy)-1,1'-binaphth-2-yloxy)-6-(4-(*tert*-butoxycarbonylamino)butyl)-1,4,7-triaza-2,5,8-trioxononan)cyclohexanecarboxylate (8c).** To a solution of the amine **6c** (140 mg, 0.213 mmol) in CH_3_CN (10 mL) at rt was added HOBt (1.2 equiv), EDCI (1.2 equiv) and the binaphthyl acid **7** [8] (122 mg, 0.190 mmol). The mixture was stirred for ca. 3 h. The solvent was then removed under reduced pressure and the resulting residue subjected to silica gel chromatography (MeOH/CH_2_Cl_2_ 1:99–4:96 as the eluent) to yield **8c** as a white solid (163 mg, 67%). [α]_D_^24^ −18.6 (*c* 2.0, MeOH); ^1^H NMR (300 MHz, CDCl_3_) δ 0.49 (d, *J =* 6.4 Hz, 3H), 0.54 (d, *J =* 6.4 Hz, 3H), 0.66–0.84 (m, 2H), 0.85–1.03 (m, 2H), 1.05–1.62 (m, 16H), 1.29 (s, 6H), 1.44 (s, 9H), 1.64–1.88 (m, 4H), 1.89–2.14 (m, 1H), 2.09 (s, 3H), 2.54–2.64 (m, 2H), 2.55 (s, 3H), 2.57 (s, 3H), 2.84–2.96 (m, 2H), 2.97–3.22 (m, 2H), 3.76–3.94 (m, 1H), 4.00–4.07 (m, 2H), 4.36 and 4.54 (ABq, *J =* 14.6 Hz, 2H), 4.39–4.48 (m, 1H), 4.78–4.82 (m, NH), 5.06 (s, 2H), 6.14 (br s, NH) 6.36 (br s, NH), 7.11–7.46 (m, 12H), 7.44 (d, *J =* 9.1 Hz, 1H), 7.84 (d, *J =* 8.9 Hz, 1H), 7.86 (d, *J =* 7.9 Hz, 1H), 7.92 (d, *J =* 8.8 Hz, 1H), 7.95 (d, *J =* 7.6 Hz, 1H); ESIMS *m*/*z*: [M + H]^+^ 1280.3 (100%); HRMS–ESI *m*/*z*: [M + H]^+^ calcd for C_72_H_94_N_7_O_12_S, 1280.6676; found, 1280.6627.

**Benzyl 1-((3*R*,6*R*)-6-aminobutyl-3-(3-guanidinopropyl)-9-((*S*)-2'-(3-methylbutoxy)-1,1'-binaphth-2-yloxy)-1,4,7-triaza-2,5,8-trioxononan)cyclohexanecarboxylate dihydrochloride (2c).** The protected amine **8c** (106 mg, 0.083 mmol) in 1:1 CH_2_Cl_2_/TFA (6 mL/0.10 mmol) solution was stirred for 12 h at rt. The solvent was removed under reduced pressure, and the residue was resuspended in a minimal volume of methanol. The solution was then treated with an excess of 2 M HCl/diethyl ether solution (2 mL, 0.01 mmol) and the solvent evaporated. The crude product was purified by precipitation from MeOH by addition of diethyl ether to yield **2c** as an off white solid (35 mg, 43%). [α]_D_^24^ −10.9 (*c* 3.4, MeOH); ^1^H NMR (300 MHz, CD_3_OD) δ 0.50 (d, *J =* 6.4 Hz, 3H), 0.56 (d, *J =* 6.4 Hz, 3H), 0.84–1.02 (m, 2H), 1.03–3.15 (m, 21H), 2.68–2.92 (m, 2H), 2.96–3.20 (m, 2H), 3.91–3.98 (m, 1H), 4.09–4.16 (m, 2H), 4.24–4.36 (m, 1H), 4.42 and 4.54 (ABq, *J =* 14.6 Hz, 2H), 5.02 and 5.09 (ABq, *J =* 12.3 Hz, 2H), 7.06 (t, *J* = 5.5 Hz, 2H), 7.17–7.22 (m, 2H), 7.27–7.36 (m, 7H), 7.44 (d, *J =* 8.8 Hz, 1H), 7.53 (d, *J =* 9.1 Hz, 1H), 7.90 (t, *J* = 7.0 Hz, 2H), 8.00 (dd, *J* = 2.3 and 9.1 Hz, 2H), 8.09 (s, NH); ^13^C NMR (75 MHz, CD_3_OD) δ 22.5, 22.6, 22.8, 23.0, 25.6, 26.2, 26.3, 27.7, 30.0, 32.3, 32.9, 33.7, 39.3, 40.3, 42.0, 53.3, 54.3, 60.3, 67.8, 69.0, 69.1, 115.9, 116.9, 120.5, 121.7, 124.8, 125.2, 125.9, 126.4, 127.5, 127.6, 129.1, 129.2, 129.6, 130.7, 130.9, 131.4, 135.0, 135.2, 137.4, 154.0, 155.9, 158.5, 170.7, 173.1, 173.6, 175.4; ESIMS *m*/*z*: [M + H]^+^ 915.0 (10%), [M + 2H]^2+^ 457.9 (100); HRMS–ESI *m*/*z*: [M + H]^+^ calcd for C_53_H_68_N_7_O_7_, 914.5175; found, 914.5130 (100%). HPLC analysis of this compound was also undertaken with a gradient system comprised of H_2_O containing 10% CH_3_CN and 0.1% TFA 90:10:0.5 (A), and CH_3_CN containing 0.1% TFA (B). The gradient profile was 0–3 min, linear gradient 0 to 50% B; 4–13 min, linear gradient 50 to 80% of B; 14–15 min, linear gradient 80 to 100% B; *t*_R_ = 6.1 min, 96% pure.

### Determination of minimum inhibitory concentration (MIC)

MIC studies (Table 1) were performed on *Staphylococcus aureus* wild type (ATCC 6538P), and *Staphylococcus epidermidis* (ATCC 12228) in Mueller–Hinton broth (Oxoid Ltd, England) supplemented with 50 mg/L CaCl_2_. As in [6], MIC determinations for clinical isolates of *Enterococcus faecium* were conducted by growth in Enterococcosal broth (Becton Dickinson Microbiology Systems). Briefly, overnight stationary phase cultures were diluted 1:1000 into fresh media and then incubated with two-fold dilutions of compounds in media, typically with a highest concentration of 128 µg/mL, in a 96-well plate. Plates were incubated overnight at 37 °C and the MIC was recorded as the highest concentration at which bacterial growth was observed.

Compound **2c** (Table 2) was tested at JMI Laboratories through Ordway Research Institute (USA) on *Staphylococcus aureus* (MSSA; MRSA; VISA), *S. epidermidis* and *E. faecium* (VRE and VSE). The microdilution reference methods (M7-A6 (2003) and M11-A6 (2004)) of the CLSI/NCCLS were used. Quality control ranges for the selected control agent vancomycin from CLSI/NCCLS M100-S15 (2005) were used. Panels were produced in volumes of 100 µL/well over a concentration of 0.03–32 µg/mL. A growth control was included for each dilution series. MIC results were produced by using the M7-A6 and M11-A6 CLSI/NCCLS procedures in cation-adjusted Mueller–Hinton broth media with supplements as required for the test species. The compounds were initially dissolved in DMSO and then diluted.

## Supporting Information

File 1 Experimental procedures and associated spectroscopic data (NMR and MS) for the syntheses of compounds **2a**–**b** and **2d**–**g**.
