# Supplementary material for: Binaphthyl-anchored antibacterial tripeptide derivatives with hydrophobic C-terminal amino acid variations
Source: Beilstein J Org Chem. 2012 Aug 9;8:1265–70. doi: 10.3762/bjoc.8.142 (PMC3458747; doi:10.3762/bjoc.8.142)
Supplement: File 1 — Experimental procedures and associated spectroscopic data (NMR and MS) for the syntheses of compounds 2a–b and 2d–g. [file Beilstein_J_Org_Chem-08-1265-s001.pdf]

Supporting Information
for
Binaphthyl-anchored antibacterial tripeptide derivatives with hydrophobic C-terminal amino acid variations

John B. Bremner*[1], Paul A. Keller*[1], Stephen G. Pyne*[1], Mark J. Robertson[1], K. Sakthivel[1], Kittiya Somphol[1], Dean Baylis[2], Jonathan A. Coates[2], John Deadman[3], Dharshini Jeevarajah[2] and David I. Rhodes[4]

Address: [1]School of Chemistry, University of Wollongong, Wollongong, NSW 2522, Australia; [2]Avexa Ltd, 576 Swan St, Richmond, Vic 3121, Australia; [3]Chemocopeia Pty Ltd, 114 Kay St, Carlton, Melbourne, Vic 3053, Australia and [4]JDJ Bioservices Pty Ltd, 576 Swan St, Richmond, Vic 3121, Australia

Email: John B. Bremner* - jbremner@uow.edu.au; Paul A. Keller* - keller@uow.edu.au; Stephen G. Pyne* - spyne@uow.edu.au

*Corresponding author

Experimental procedures and associated spectroscopic data (NMR and MS) for the syntheses of compounds 2a–b and 2d–g

1. General notes

Electrospray ionization (ESI) mass spectra were obtained on a VG Autospec spectrometer. High-resolution mass spectra (HRMS) were determined on a micromass QTof2 spectrometer with polyethylene glycol or polypropylene glycol as the internal standard. The *m*/*z* values are stated with their peak intensity as a percentage in parentheses. Optical rotations were measured by using a Jasco polarimeter in a 10 mm path length cell. Proton and carbon nuclear magnetic resonance (NMR) spectra were obtained as specified on a Varian Mercury 300 MHz, Varian Inova 500 MHz or Varian Venus 500 MHz spectrometer. Spectra were recorded in the specified deuterated solvent, and referenced to the residual non-deuterated solvent signal. Chemical shifts (δ) in ppm were measured relative to the internal reference.

Proton and carbon assignments were determined through the interpretation of two dimensional spectra (COSY, gHSQC, gHMBC, ROESY, and TOCSY). Analytical thin layer chromatography (TLC) was carried out on Merck silica gel 60 F_{254} precoated aluminium plates with a thickness of 0.2 mm. All column chromatography was performed under "flash" conditions on Merck silica gel 60 (230–400 mesh). Chromatography solvent mixtures were measured by volume. Analytical HPLC was performed on a Waters instrument equipped with a 1525 binary pump and a 2487 detector. Chromatographic separation was achieved with a C18 column (150 × 4.60 mm, 5 µm; Phenomenex), injection volume 20 µL, flow rate 1.0 mL/min and UV detection at 254 nm. Solvent was removed under reduced pressure with a Büchi rotary evaporator. Solvents were purified and dried based on standard techniques. All compounds were judged to be of greater than 95% purity based upon ^{1}H NMR and TLC analysis. Compounds for antibacterial testing were of >96% purity by HPLC analysis. Starting materials and reagents were purchased from Sigma-Aldrich Pty Ltd, Auspep Pty Ltd or Bachem Ltd and were used as received.

2. General synthetic procedures [1]

Protocol 1: Peptide coupling

To a solution of the acid in dichloromethane or acetonitrile (10 mL/0.10 mmol) at rt was added EDCI (1.2 equiv), HOBt (1.2 equiv), and the amine (1 equiv). When the amine was a hydrochloride salt, DIPEA (1.2 equiv) was also added. After stirring of the mixture for 1–3 h, the solvent was removed under reduced pressure, and then the resulting residue was subjected to silica gel column chromatography (MeOH/CH_2Cl_2 1–4:99–96 as the eluent) to afford a coupled product.

Protocol 2: *N*-Fmoc deprotection

The Fmoc-protected amine was stirred in 1 equiv of piperidine/acetonitrile (5 mL/0.10 mmol) overnight at rt, unless otherwise stated. The solvent was removed under reduced pressure, and the residue was subjected to column chromatography with MeOH/CH_2Cl_2 2:98 and then MeOH/CH_2Cl_2 5–15:95–85 to yield the free amine.

Protocol 3: *N*-Boc, Pbf and Pmc deprotection

The *N*-Boc-, Pbf- or Pmc-protected amine was stirred for 1 h (for Boc) or overnight (for Pbf and Pmc) in CH_2Cl_2/TFA 1:1 (6 mL/0.10 mmol) solution at rt. The solvent was removed under reduced pressure, and the residue was resuspended in a minimal volume of methanol or CH_2Cl_2. The solution was then treated with an excess of 2 M HCl/ether (2 mL/0.01 mmol)

solution and the solvent evaporated. The product was purified by precipitation from CH_2Cl_2 or MeOH and diethyl ether.

3. Protecting group abbreviations

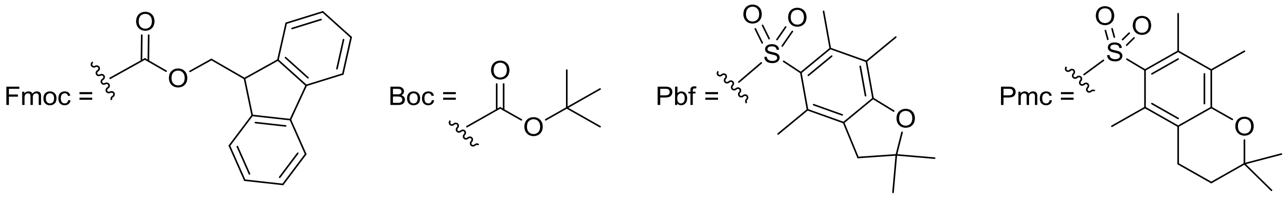

Benzyl 1-(*tert*-butoxycarbonylamino)cyclobutanecarboxylate

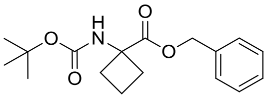

To Boc-1-amino-1-cyclobutane carboxylic acid (200 mg, 0.93 mmol) and potassium carbonate (440 mg, 3.18 mmol) in acetone (20 mL) was added benzyl bromide (0.12 mL, 1.01 mmol). The resulting solution was heated under reflux overnight before being cooled, filtered and evaporated to dryness. The resulting residue was subjected to flash column chromatography over silica, eluting with ethyl acetate/hexane 5:95 to first remove benzyl bromide, then with CH_2Cl_2 to yield the titled product (262 mg, 92%) as a white solid. ^{1}H NMR (300 MHz, $CDCl_3$) δ 1.39 (s, 9H), 1.90–2.09 (m, 2H), 2.12–2.39 (m, 2H) 2.51–2.67 (m, 2H), 5.18 (s, 2H), 5.40 (s, 1H, NH), 7.25–7.40 (m, 5H); ^{13}C NMR (75 MHz, $CDCl_3$) δ 15.0, 28.1, 31.2, 58.2, 66.7, 79.6, 127.9, 128.0, 128.3, 135.7, 154.7, 173.6; ESIMS (*m*/*z*): $[M + Na]^+$ 1204 (80%), $[M + H]^+$ 1182 (100).

Benzyl 1-amino-cyclobutanecarboxylate (4a)

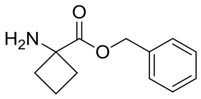

This compound was prepared following protocol 3 and basification, by using the above protected amino acid (110 mg, 0.36 mmol) to yield the titled product (67 mg, 91%) as a pale yellow oil. ^{1}H NMR (300 MHz, $CDCl_3$) δ 1.79–2.10 (m, 6H/NH_2), 2.49–2.65 (m, 2H), 5.18 (s, 2H), 7.25–7.41 (m, 5H); ^{13}C NMR (75 MHz, $CDCl_3$) δ 14.1, 34.0, 58.4, 66.5, 127.9, 128.1, 128.4, 135.8, 175.8; ESIMS (*m*/*z*): $[M + H]^+$ 206 (100%).

Benzyl 1-((3*R*)-1-aza-6-(2,3-dihydro-2,2,4,6,7-pentamethyl-2*H*-1-benzofuran-5-yl)sulfonyl]guanidino)-3-(9*H*-9-fluorenylmethoxycarbonylamido)-2-oxohexanyl)cyclobutanecarboxylate (5a)

This compound was prepared following protocol 1, by using Fmoc-(*R*)-Arg(Pbf)-OH (150 mg, 0.23 mmol) and **4a** (67 mg, 0.33 mmol) to yield the Fmoc-protected product (185 mg, 96%) as a white foam. ^{1}H NMR (300 MHz, $CDCl_3$) δ 1.37 (s, 6H), 1.50–1.61 (m,

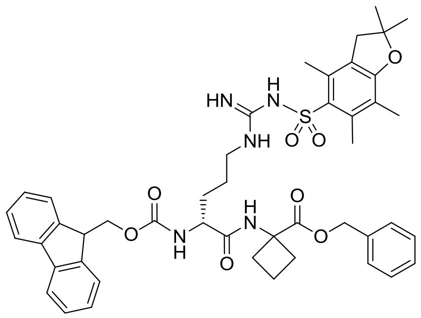

3H), 1.80–2.00 (m, 3H), 2.03 (s, 3H), 2.09–2.32 (m, 2H), 2.50 (s, 3H), 2.55–2.74 (m, 2H), 2.58 (s, 3H), 2.84 (s, 2H), 3.13–3.21 (m, 2H), 4.05–4.10 (m, 1H), 4.25–4.28 (m, 2H), 4.26–4.60 (m, 1H), 5.08 (s, 2H), 6.17 (d, J = 7.8 Hz, 2H, 2NH), 6.36 (s, 2H, NH), 7.16–7.34 (m, 9H), 7.51 (d, J = 7.2 Hz, 2H), 7.69 (d, J = 7.5 Hz, 2H), 7.93 (br s, 1H, NH); ^{13}C NMR (75 MHz, $CDCl_3$) δ 12.3, 15.4, 17.9, 19.2, 25.2, 28.4, 31.1, 31.3, 43.0, 46.9, 53.3, 53.9, 58.3, 64.8, 66.85, 66.9, 86.2, 117.4, 119.8, 124.6, 125.0, 126.8, 126.9, 127.3, 127.5, 127.7, 128.0, 128.28, 128.3, 132.1, 132.6, 135.5, 138.2, 141.03, 141.05, 143.5, 143.7, 156.3, 156.4, 158.6, 172.0, 173.1; ESIMS (*m*/*z*): $[M + H]^+$ 836 (100%).

Benzyl 1-((3*R*)-3-amino-1-aza-6-(2,3-dihydro-2,2,4,6,7-pentamethyl-2*H*-1-benzofuran-5-yl)sulfonyl]guanidino)-2-oxohexan)cyclobutanecarboxylate (6a)

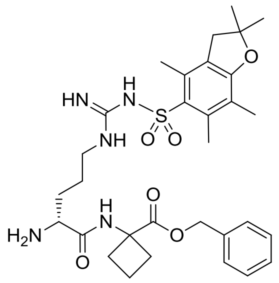

This compound was prepared following protocol 2, by using **5a** (185 mg, 0.22 mmol) to yield the product **6a** (111 mg, 82%). ^{1}H NMR (300 MHz, $CDCl_3$) δ 1.46–3.51 (m, 29H), 5.14–5.44 (m, 3H), 6.20–6.70 (m, 3H, 3NH), 7.32–7.44 (m, 5H), 8.08–8.19 (m, 1H, NH); ^{13}C NMR (75 MHz, $CDCl_3$) δ 12.5, 15.4, 18.0, 19.3, 25.3, 26.6, 28.6, 31.5, 40.7, 43.2, 54.0, 58.1, 67.0, 86.4, 114.5, 124.6, 127.9, 128.5, 132.1, 132.9, 135.6, 138.3, 156.4, 158.7, 173.2; ESIMS (*m*/*z*): $[M + H]^+$ 614 (100%); HRMS–ESI *m*/*z*: calcd for $C_{31}H_{44}N_5O_6S$ 614.3012, found 614.3034.

Benzyl 1-((3*R*,6*R*)-3-(3-[(2,3-dihydro-2,2,4,6,7-pentamethyl-2*H*-1-benzofuran-5-yl)sulfonyl]guanidinopropyl)-9-((*S*)-2'-(3-methylbutoxy)-1,1'-binaphth-2-yloxy)-6-(4-(*tert*-butoxycarbonylamino)butyl)-1,4,7-triaza-2,5,8-trioxononan)cyclobutanecarboxylate (8a)

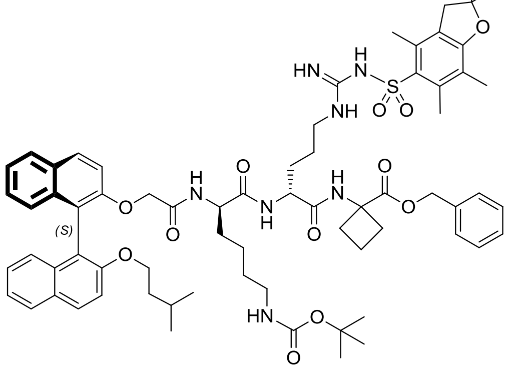

This compound was prepared following protocol 1, by using **7** (100 mg, 0.16 mmol) and **6a** (111 mg, 0.18 mmol) to yield the product (30 mg, 16%) as an off white solid. ^{1}H NMR (300 MHz, $CDCl_3$) δ 0.48 (d, J = 6.0 Hz, 3H), 0.53 (d, J = 6.0 Hz, 3H), 0.74–0.88 (m, 2H), 0.94–0.99 (m, 1H), 1.04–1.58 (m, 8H), 1.42 (s, 9H), 1.44 (s, 6H), 1.67–1.82 (m, 1H), 1.74–2.11 (m, 4H), 2.07 (s, 3H), 2.25–2.38 (m, 2H), 2.49 (s, 3H), 2.57 (s, 3H), 2.66–2.74 (m, 1H), 2.91 (s, 2H), 2.92–3.08 (m, 2H), 3.83–3.90 (m, 1H),

3.98–4.05 (m, 2H), 4.38 and 4.54 (ABq, J = 14.7 Hz, 2H), 4.40–4.51 (m, 1H), 4.69–4.79 (m, 1H, NH), 6.14–6.19 (m, 3H, 3NH), 7.10–7.38 (m, 12H), 7.43 (d, J = 9.3 Hz, 1H), 7.65 (br s, 1H, NH), 7.83–7.87 (m, 2H), 7.94 (dd, J_1 = 9.0 Hz, J_2 = 2.7 Hz, 2H); ^{13}C NMR (75 MHz, $CDCl_3$) δ 12.5, 15.6, 18.0, 19.3, 22.0, 22.3, 22.5, 24.5, 25.2, 28.4, 28.6, 29.1, 29.7, 31.2, 31.4, 37.9, 40.1, 40.4, 43.2, 58.4, 66.8, 68.0, 68.2, 78.9, 86.3, 114.3, 115.8, 117.5, 119.47, 120.4, 123.9, 124.2, 124.6, 124.9, 125.5, 126.5, 126.7, 127.8, 127.9, 128.1, 128.5, 129.2, 129.7, 129.8, 132.2, 132.9, 133.6, 133.8, 135.9, 138.3, 152.2, 154.4, 156.0, 156.3, 158.7, 169.2, 171.1, 171.3, 173.0; ESIMS (*m/z*): $[M + Na]^+$ *m/z* 1261 (100%); $[M + H]^+$ 1239 (65); HRMS–ESI *m/z*: calcd for $C_{69}H_{88}N_7O_{12}S$ 1238.6212, found 1238.6194.

Benzyl 1-((3*R*,6*R*)-6-butylamino-3-(3-guanidinopropyl)-9-((*S*)-2'-(3-methylbutoxy)-1,1'-binaphth-2-yloxy)-1,4,7-triaza-2,5,8-trioxononan)cyclobutanecarboxylate dihydrochloride (2a)

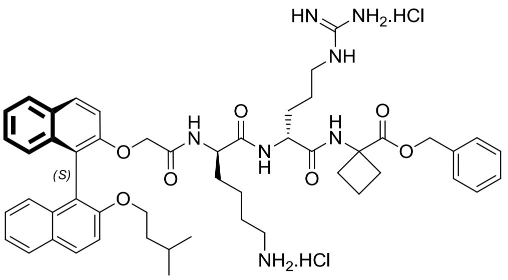

This compound was prepared following protocol 3, by using **8a** (30 mg, 0.024 mmol) to yield the product (22 mg, 96%) as a pale yellow solid. ^{1}H NMR (500 MHz, CD_3OD) δ 0.55 (d, J = 6.5 Hz, 3H), 0.58 (d, J = 7.0 Hz, 3H), 0.92–1.05 (m, 2H), 1.11–1.30 (m, 4H), 1.47–1.70 (m, 7H), 1.99–2.05 (m, 2H), 2.01–2.26 (m, 1H), 2.29–2.35 (m, 1H), 2.54–2.59 (m, 1H), 2.68–2.73 (m, 1H), 2.72–2.87 (m, 2H), 3.05–3.11 (m, 2H), 3.94–3.99 (m, 1H), 4.11–4.16 (m, 2H), 4.23–4.26 (m, 1H), 4.47 and 4.55 (ABq, J = 14.5 Hz, 2H), 5.12 and 5.15 (ABq, J = 12.5 Hz, 2H), 7.06 (dd, J_1 = 14.5 Hz, J_2 = 8.5 Hz, 2H), 7.20–7.24 (m, 2H), 7.30–7.37 (m, 7H), 7.47 (d, J = 9.0 Hz, 1H), 7.55 (d, J = 8.5 Hz, 1H), 7.90 (d, J = 8.0 Hz, 2H), 8.02 (dd, J_1 = 9.0 Hz, J_2 = 4.0 Hz, 2H); ^{13}C NMR (75 MHz, CD_3OD) δ 16.4, 22.6, 22.8, 23.0, 25.6, 26.1, 27.7, 30.0, 32.1, 32.3, 39.3, 40.3, 42.0, 53.6, 54.2, 59.6, 68.0, 69.0, 69.2, 116.0, 116.9, 120.6, 121.8, 124.8, 125.2, 126.0, 126.4, 127.6, 129.2, 129.3, 129.6, 130.9, 131.4, 135.1, 135.3, 137.4, 154.1, 156.0, 158.6, 173.3, 173.7, 174.4; ESIMS (*m/z*): $[M + H]^+$ 886 (80%), $[M + 2H]^{2+}$ 443 (100); HRMS–ESI *m/z*: calcd for $C_{51}H_{64}N_7O_7$ 886.4867, found 886.4881.

Benzyl 1-(*tert*-butoxycarbonylamino)cyclopentanecarboxylate

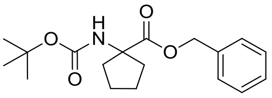

To 1-(*tert*-butyloxycarbonyl)cyclopentanecarboxylic acid (120 mg, 0.523 mmol) and potassium carbonate (178 mg, 1.29 mmol) in acetone (25 mL) was added benzyl bromide (0.1 mL, 0.92 mmol). The resulting solution was heated

under reflux overnight before being cooled, filtered and evaporated to dryness. The residue was purified by column chromatography, eluting with 5% ethyl acetate/hexane to first remove benzyl bromide, then with CH_2Cl_2 to yield the title product (173 mg, 96%) as a white solid. ^{1}H NMR (500 MHz, $CDCl_3$) δ 1.37 (s, 9H), 1.72–1.75 (m, 4H), 1.81–1.98 (m, 2H), 2.18–2.23 (m, 2H), 5.08 (br s, 1H, NH), 5.13 (s, 2H), 7.26–7.32 (m, 5H); ^{13}C NMR (75 MHz, $CDCl_3$) δ 24.4, 28.1, 37.5, 66.7, 78.8, 127.9, 127.95, 128.3, 135.8, 155.0, 174.4; ESIMS (*m*/*z*): $[M + H]^+$ 320 (50%), $[M - C_4H_8]^+$ 264 (100); $[M - Boc]^+$ 220 (100)

Benzyl 1-aminocyclopentanecarboxylate (4b)

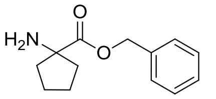

Compound **4b** [2] was prepared following protocol 3 and basification, by using the above amino acid derivative (170 mg, 0.532 mmol) to yield the title product (102 mg, 87%) as a pale yellowish oil. ^{1}H NMR (500 MHz, $CDCl_3$) δ 1.50–1.61 (m, 2H), 1.65–1.79 (m, 2H), 1.80–1.91 (m, 2H), 2.02–2.18 (m, 2H), 5.13 (s, 2H), 7.30–7.34 (m, 5H); ^{13}C NMR (126 MHz, $CDCl_3$) δ 24.9, 39.4, 64.8, 66.5, 127.8, 128.0, 128.4, 135.9, 177.8; ESIMS (*m*/*z*): $[M + H]^+$ 220 (100%); HRMS–ESI *m*/*z*: calcd for $C_{13}H_{18}NO_2$ 220.1338, found 220.1348.

Benzyl 1-((3*R*)-1-aza-6-(2,3-dihydro-2,2,4,6,7-pentamethyl-2*H*-1-benzofuran-5-yl)sulfonylguanidino)-3-(9*H*-9-fluorenylmethoxycarboxamido)-2-oxohexan)cyclopentanecarboxylate (5b)

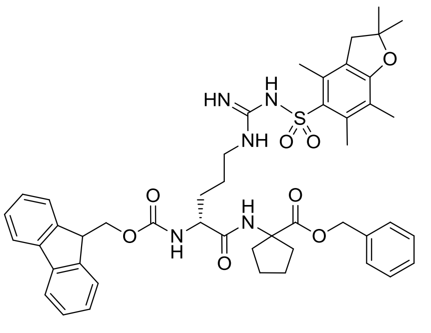

This compound was prepared following protocol 1, Fmoc-(*R*)-Arg(Pbf)-OH (296 mg, 0.456 mmol) and **4b** (100 mg, 0.456 mmol) to yield **5b** (241 mg, 61%) as a white foam. ^{1}H NMR (500 MHz, $CDCl_3$) δ 1.41 (s, 6H), 1.47–1.75 (m, 7H), 1.77–1.87 (m, 1H), 1.95–2.16 (m, 3H), 2.05 (s, 3H), 2.24–2.27 (m, 1H), 2.50 (s, 3H), 2.57 (s, 3H), 2.88 (s, 2H), 3.11–3.23 (m, 2H), 3.89–4.11 (m, 1H), 4.27–4.28 (m, 3H), 5.04–5.09 (m, 2H), 6.06 (br s, 1H, NH), 6.29 (s, 2H, 2NH), 7.20–7.37 (m, 5H), 7.52 (d, *J* = 7.5 Hz, 2H), 7.71 (d, *J* = 7.5 Hz, 2H); ESIMS (*m*/*z*): $[M + H]^+$ 850 (100%).

Benzyl 1-((3*R*)-3-amino-1-aza-6-(2,3-dihydro-2,2,4,6,7-pentamethyl-2*H*-1-benzofuran-5-yl)sulfonyl]guanidino)-2-oxohexan)cyclopentanecarboxylate (6b)

This compound was prepared following protocol 2, by using **5b** (241 mg, 0.279 mmol) to yield **6b** (152 mg, 85%). ^{1}H NMR (500 MHz, $CDCl_3$) δ 1.44 (s, 6H), 1.42–2.11 (m,

12H/NH_2), 1.44 (s, 6H), 2.07 (s, 3H), 2.16–2.29 (m, 2H), 2.57 (s, 3H), 2.63 (s, 3H), 2.93 (s, 2H), 3.13–3.21 (m, 2H), 3.40–3.43 (m, 1H), 3.98–4.06 (m, 1H), 5.05 and 5.09 (ABq, *J* = 12.5 Hz, 2H), 6.37–6.49 (m, 3H, 3NH), 7.45–7.31 (m, 5H), 7.52 (s, 1H, NH); ^{13}C NMR (126 MHz, $CDCl_3$) δ 12.4, 17.9, 19.2, 23.2, 24.4, 28.5, 30.8, 37.0, 40.1, 43.1, 53.4, 65.4, 66.9, 86.2, 117.4, 124.5, 128.0, 128.1, 128.4, 132.1, 135.6, 138.1, 156.4, 158.6, 168.4, 173.4; ESIMS (*m*/*z*): [M + H]$^+$ 628 (100%); HRMS–ESI *m*/*z*: calcd for $C_{32}H_{46}N_5O_6S$ 628.3169, found 628.3182.

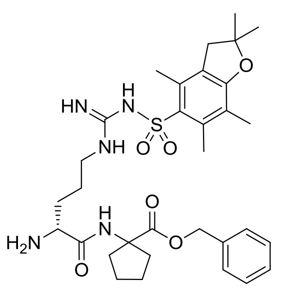

Benzyl 1-((3*R*,6*R*)-3-(3-[(2,3-dihydro-2,2,4,6,7-pentamethyl-2*H*-1-benzofuran-5-yl)sulfonyl]guanidinopropyl)-9-((*S*)-2'-(3-methylbutoxy)-1,1'-binaphth-2-yloxy)-6-(4-(*tert*-butoxycarbonylamino)butyl)-1,4,7-triaza-2,5,8-trioxononan)cyclopentanecarboxylate (8b)

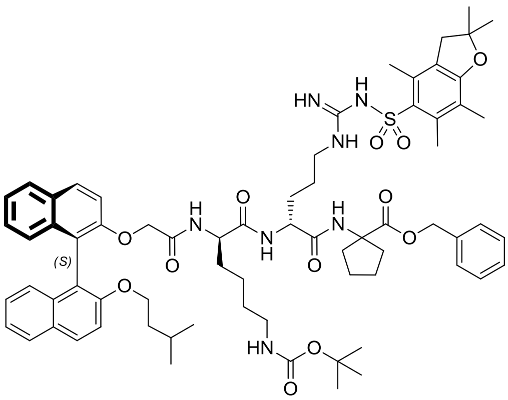

This compound was prepared following protocol 1, by using **7** (100 mg, 0.155 mmol) and **6b** (141 mg, 0.224 mmol) to yield the title product (123 mg, 63%) as a white solid. ^{1}H NMR (500 MHz, $CDCl_3$) δ 0.47 (d, *J* = 6.4 Hz, 3H), 0.52 (d, *J* = 6.4 Hz,3H), 0.75–0.82 (m, 2H), 0.88–1.02 (m, 1H), 1.06–1.54 (m, 10H), 1.42 (s, 9H), 1.43 (s, 3H), 1.44 (s, 3H), 1.59–1.81 (m, 4H), 1.95–1.97 (m, 2H), 2.07 (s, 3H), 2.11–2.15 (m, 1H), 2.24–2.30 (m, 1H), 2.50 (s, 3H), 2.57 (s, 3H), 2.81–2.99 (m, 2H), 2.91 (s, 2H), 3.00–3.21 (m, 2H), 3.84–3.87 (m, 1H), 4.01–4.05 (m, 2H), 4.38 (d, *J* = 14.5 Hz, 2H), 4.54 (d, *J* = 15.0 Hz, 1H), 5.07 (s, 2H), 6.15 (d, *J* = 6.9 Hz, 1H, NH), 6.25 (br s, 2H, 2NH), 7.11 (d, *J* = 8.5 Hz, 1H), 7.15 (d, *J* = 8.4 Hz, 1H), 7.21–7.36 (m, 10H), 7.43 (d, *J* = 9.1 Hz, 1H), 7.84 (d, *J* = 8.4 Hz, 1H), 7.85 (d, *J* = 8.4 Hz, 1H), 7.93 (d, *J* = 6.9 Hz, 1H), 7.94 (d, *J* = 9.0 Hz, 1H); ^{13}C NMR (126 MHz, $CDCl_3$) δ 12.4, 17.9, 19.3, 22.0, 22.2, 22.4, 23.2, 24.4, 24.46, 24.5, 25.3, 28.4, 28.5, 29.1, 31.1, 36.9, 37.1, 37.9, 40.0, 43.2, 52.8, 53.4, 65.9, 66.8, 67.9, 68.2, 78.8, 86.3, 114.2, 115.8, 117.4, 119.3, 120.3, 123.8, 124.1, 124.5, 124.9, 125.4, 126.5, 126.6, 127.8, 127.9, 128.0, 128.4, 129.1, 129.7, 132.1, 132.9, 133.5, 133.8, 135.8, 138.2, 152.1, 154.3, 156.0, 156.3, 158.6, 169.1, 171.3, 173.8; ESIMS (*m*/*z*): [M + Na]$^+$ 1275 (20%); [M + H]$^+$ 1253 (100).

Benzyl 1-((3*R*,6*R*)-6-butylamino-3-(3-guanidinopropyl)-9-((*S*)-2'-(3-methylbutoxy)-1,1'-binaphth-2-yloxy)- 1,4,7-triaza-2,5,8-trioxononan)cyclopentanecarboxylate dihydrochloride (2b)

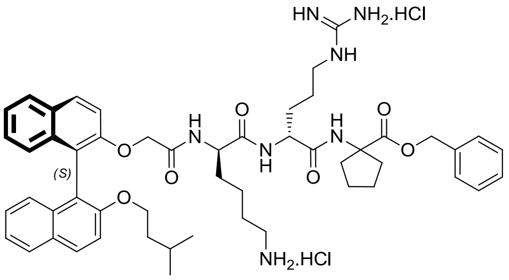

This compound was prepared following protocol 3, by using **8b** (110 mg, 0.088 mmol) to yield **2b** (83 mg, 97%) as an off-white solid. ^{1}H NMR (500 MHz, CD_3OD) δ 0.51 (d, *J* = 6.5 Hz, 3H), 0.57 (d, *J* = 6.5 Hz, 3H), 0.94–0.97 (m, 2H), 1.12–1.18 (m, 2H), 1.20–1.28 (m, 2H), 1.42–1.44 (m, 1H), 1.54–1.64 (m, 5H), 1.66–1.85 (m, 5H), 1.97–2.00 (m, 2H), 2.10–2.13 (m, 1H), 2.29–2.32 (m, 1H), 2.78–2.79 (m, 2H), 3.07–3.08 (m, 2H), 3.93–3.98 (m, 1H), 4.11–4.16 (m, 2H), 4.25–4.28 (m, 1H), 4.45 and 4.55 (ABq, *J* = 14.7 Hz, 2H), 5.07 and 5.12 (ABq, *J* = 12.3 Hz, 2H), 7.07 (t, *J* = 9.3 Hz, 2H), 7.19–7.22 (m, 2H), 7.29-7.35 (m, 7H), 7.47 (d, *J* = 8.9 Hz, 1H), 7.55 (d, *J* = 9.0 Hz, 1H), 7.89 (d, *J* = 8.3 Hz, 1H), 7.92 (d, *J* = 8.3 Hz, 1H), 8.01 (d, *J* = 8.9 Hz, 1H), 8.02 (d, *J* = 8.9 Hz, 1H); ^{13}C NMR (126 MHz, CD_3OD) δ 22.6, 22.8, 23.0, 25.4, 25.6, 26.1, 27.7, 30.0, 32.2, 37.6, 38.0, 39.3, 40.4, 41.9, 53.4, 54.0, 67.2, 68.0, 69.0, 69.2, 116.0, 116.9, 120.5, 121.8, 124.8, 125.2, 125.9, 126.4, 127.5, 127.6, 129.1, 129.2, 129.25, 129.6, 130.7, 130.9, 131.4, 135.0, 135.2, 137.3, 154.0, 155.9, 158.5, 170.7, 173.0, 173.7, 175.1; ESIMS (*m*/*z*): $[M + H]^+$ 900 (5%); 451 $[M + 2H]^{2+}$ (100); HRMS–ESI *m*/*z*: calcd for $C_{52}H_{66}N_7O_7$ 900.5024, found 900.5042.

Benzyl 4-(*tert*-butoxycarbonylamino)tetrahydro-2*H*-pyran-4-carboxylate

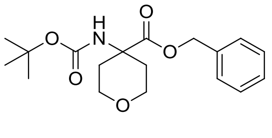

Benzyl bromide (0.25 mL, 2 mmol) was added to a stirred suspension of 4-(*tert*-butoxycarbonylamino)tetrahydro-2*H*-pyran-4-carboxylic acid (245 mg, 1.0 mmol) and anhydrous potassium carbonate (345 mg, 2.5 mmol) in anhydrous THF (30 mL). The mixture was heated under reflux for 3 h. The mixture was cooled and poured into water (50 mL) and extracted with EtOAc (3 × 50 mL). The combined organic layers were washed with sat. $NaHCO_3$ and sat. NaCl solution and dried with anhydrous sodium sulfate. After evaporation of organic solvent, crude product was purified by column chromatography on silica gel using EtOAc/petroleum ether (0–20:100–80) to give the benzyl ester (230 mg, 69%). ^{1}H NMR (500 MHz, $CDCl_3$) δ1.38 (s, 9H), 1.79–1.85 (m, 2H), 2.17–2.20 (m, 2H), 3.61–3.64 (m, 2H), 3.76–3.79 (m, 2H), 4.70 (bs, 1H), 5.16 (s, 2H), 7.32–7.41 (m, 5H); ESIMS (*m*/*z*): $[M + H]^+$ 336 (30%).

Benzyl 4-aminotetrahydro-2*H*-pyran-4-carboxylate (4d)

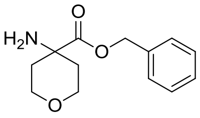

The Boc-protected compound from above (213 mg, 0.64 mmol) was added to a mixture of trifluoroacetic acid/CH_2Cl_2 3:7, 20 mL. The solution was stirred at rt for 2 h. The solvent was evaporated under the reduced pressure and the residue was redissolved in CH_2Cl_2 (5 mL), and washed with sat. aq. $NaHCO_3$, water, dried ($MgSO_4$) and filtered. The solvent was removed by rotary evaporation to give **4d** (120 mg, 80%). ^{1}H NMR (500 MHz, $CDCl_3$) δ 1.43 (d, *J* = 14.0 Hz; 2H), 1.79 (br s, 2H), 2.05–2.10 (m, 2H), 3.56–3.60 (m, 2H), 3.76–3.81 (m, 2H), 5.10 (s, 2H), 7.18–7.30 (m, 5H); ^{13}C NMR (125 MHz, CD_3OD) δ 31.7, 56.6, 62.7, 68.5, 128.6, 128.7, 128.7, 135.0, 169.8; ESIMS (*m*/*z*): $[M + H]^+$ 236 (70%).

Benzyl 4-(2-((*R*)-(9*H*-9-fluorenylmethoxycarboxamido))-5-(3-[(2,3-dihydro-2,2,4,6,7-pentamethyl-2*H*-1-benzofuran-5-yl)sulfonyl]guanidino)pentanamido)tetrahydro-2*H*-pyran-4-carboxylate (5d)

To a solution of Fmoc-(*R*)-Arg(Pbf)-OH (324 mg, 0.50 mmol) and **4d** (100 mg, 0.24 mmol) in CH_3CN (20 mL), was added EDCI·HCl (200 mg, 1.0 mmol), and HOBt (54 mg, 0.5 mmol). The resulting mixture was stirred at rt for 16 h. The reaction mixture was diluted with EtOAc (50 mL) and the solution was washed sat. aq. $NaHCO_3$, water, dried ($MgSO_4$) and filtered. The solvent was removed by rotary evaporation. The residue was subjected to flash silica gel chromatography with MeOH/CH_2Cl_2 0–3:100–97 as eluent to afford the **5d** (236 mg, 65%). ^{1}H NMR (500 MHz, $CDCl_3$) δ 1.37 (s, 6H), 1.44–1.63 (m, 3H), 1.74–1.87 (m, 1H), 1.87–2.18 (m, 4H), 2.02 (s, 3H), 2.48 (s, 3H), 2.58 (s, 3H), 2.87 (s, 2H), 3.19 (br s, 2H), 3.46–3.72 (m, 4H), 4.03–4.11 (m, 1H), 4.20–4.38 (m, 3H), 5.01–5.12 (m, 2H), 5.98 (br s, 1H, NH), 6.20 (br s, 2H, 2NH), 7.13–7.37 (m, 9H), 7.45–7.58 (m, 3H/NH), 7.73 (d, *J* = 7.5 Hz, 2H); ^{13}C NMR (125 MHz, $CDCl_3$) δ 12.8, 18.3, 19.7, 25.8, 28.8, 29.8, 32.4, 32.5, 40.8, 43.4, 47.2, 53.8, 55.0, 57.2, 63.5, 63.6, 67.4, 67.5, 86.7, 118.0, 120.2, 125.1, 125.4, 127.3, 128.0, 128.3, 128.5, 128.8, 132.5, 132.9, 135.8, 138.6, 141.5, 144.0, 144.1, 156.7, 156.8, 159.2, 172.9, 173.4; ESIMS (*m*/*z*): $[M + H]^+$ 866 (100%).

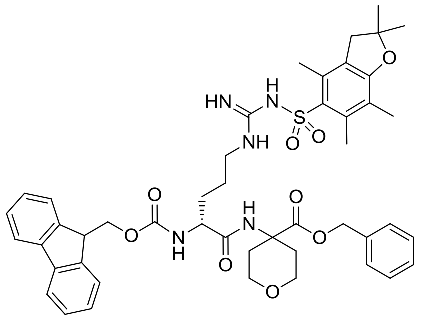

Benzyl 4-(2-((*R*)-amino)-5-(3-[(2,3-dihydro-2,2,4,6,7-pentamethyl-2*H*-1-benzofuran-5-yl)sulfonyl]guanidino)pentanamido)tetrahydro-2*H*-pyran-4-carboxylate (6d)

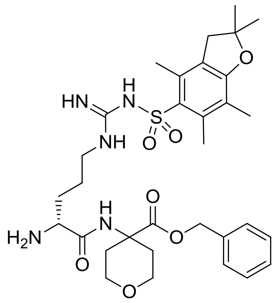

Piperidine (0.3 mL) was added to a solution of **5d** (200 mg, 0.23 mmol) in CH_3CN (20 mL). The mixture was stirred at rt for 3 h. After evaporation of solvent under reduced pressure, the residue was subjected to flash silica gel column chromatography using $MeOH/CH_2Cl_2$ 0–5:100–95 to give **6d** as a white foam (115 mg, 78%). ^{1}H NMR (500 MHz, $CDCl_3$) δ 1.37 (s, 6H), 1.48–1.50 (m, 1H), 1.63–1.66 (m, 1H), 1.84–1.92 (m, 2H), 2.00 (s, 3H), 2.03–2.13 (m, 4H), 2.42 (s, 3H), 2.49 (s, 3H), 2.86 (s, 2H), 3.06–3.09 (m, 2H), 3.38–3.40 (m, 1H), 3.50–3.57 (m, 2H), 3.66–3.69 (m, 2H), 5.01 and 5.04 (ABq, *J* = 12.5 Hz, 2H), 6.31 (br s, 1H, NH), 6.32 (br s, 2H, 2NH), 7.19–7.25 (m, 5H), 7.85 (br s, 1H, NH); ^{13}C NMR (125 MHz, $CDCl_3$) δ 12.7, 18.2, 19.6, 25.7, 28.8, 29.5, 31.9, 32.4, 32.9, 40.8, 43.5, 53.7, 54.4, 56.6, 63.6, 63.7, 67.4, 86.7, 117.8, 124.9, 128.4, 128.6, 128.8, 132.4, 133.1, 135.8, 138.5, 156.6, 159.0, 173.3, 175.5; ESIMS (*m*/*z*): $[M + H]^+$ 644 (100%).

Benzyl 4-((3*R*,6*R*)-3-(3-[(2,3-dihydro-2,2,4,6,7-pentamethyl-2*H*-1-benzofuran-5-yl)sulfonyl]guanidino)propyl)-6-(4-(*tert*-butoxycarbonylamino)butyl)-9-((*S*)-2'-(3-methylbutoxy)-1,1'-binaphth-2-yloxy)-1,4,7-triaza-2,5,8-trioxononane)tetrahydro-2*H*-pyran-4-carboxylate (8d)

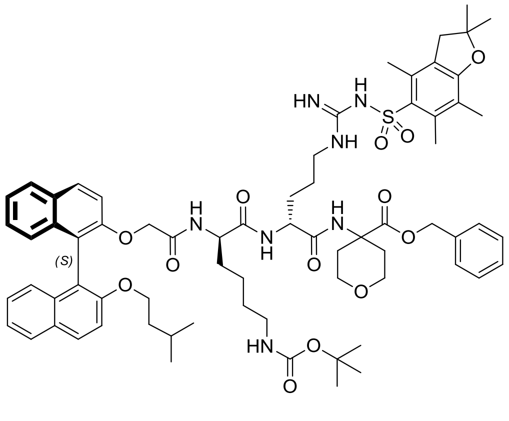

To a solution of **7** (100 mg, 0.16 mmol) and **6d** (100 mg, 0.16 mmol) in CH_3CN (20 mL), was added EDCI·HCl (100 mg, 0.50 mmol) and HOBt (54 mg, 0.50 mmol), and then the resulting mixture was stirred at rt for 16 h. The reaction mixture was diluted with EtOAc (50 mL), and the EtOAc solution was washed sat. aq. $NaHCO_3$ and water, dried ($MgSO_4$), and filtered. The solvent was removed by rotary evaporation. The residue was subjected to flash chromatography on silica gel with $MeOH/CH_2Cl_2$ 0–1:100–99 as eluent to afford **8d** (162 mg, 80%). $[\alpha]^{24}_D = -15.2$ (*c* 0.2, MeOH); ^{1}H NMR (500 MHz, $CDCl_3$) δ 0.48 (d, *J* = 6.5 Hz, 3H), 0.53 (d, *J* = 6.5 Hz, 3H), 0.67–0.86 (m, 3H), 0.87–1.02 (m, 1H), 1.03–1.60 (m, 9H), 1.42 (s, 9H), 1.429 (s, 3H), 1.433 (s, 3H), 1.89–2.22 (m, 4H), 2.06 (s, 3H), 2.48 (s, 3H), 2.56 (s, 3H), 2.79–3.21 (m, 4H), 2.90 (s, 2H), 3.60–3.64 (m, 2H), 3.69–3.71 (m, 1H), 3.75–3.77 (m, 1H), 3.85–3.90 (m, 1H), 3.89–

4.02 (m, 1H), 3.99–4.03 (m, 1H), 4.33–4.50 (m, 1H), 4.36 and 4.53 (ABq, J = 14.5 Hz, 2H), 4.71 (br s, 1H), 5.09 (s, 2H), 6.09–6.10 (m, 3H, 3NH), 7.05 (br s, 1H, NH), 7.10 (d, J = 8.5 Hz, 1H), 7.14 (d, J = 8.5 Hz, 1H), 7.20–7.36 (m, 10H), 7.43 (d, J = 9.0 Hz, 1H), 7.85 (t, J = 8.5 Hz, 2H), 7.93 (t, J = 10.0 Hz, 2H); ^{13}C NMR (125 MHz, $CDCl_3$) δ 10.2, 15.7, 17.1, 19.8, 20.1, 20.2, 22.2, 23.1, 26.2, 26.3, 27.0, 29.8, 30.1, 35.7, 37.9, 38.2, 40.1, 54.7, 61.0, 61.1, 64.7, 65.7, 66.0, 76.8, 84.1, 111.9, 113.5, 115.3, 117.1, 118.1, 121.6, 122.0, 122.4, 122.6, 123.3, 124.3, 124.5, 125.7, 125.8, 125.8, 125.9, 126.2, 126.2, 126.9, 127.5, 127.6, 129.9, 130.6, 131.3, 131.6, 133.4, 136.0, 149.8, 152.1, 153.7, 154.0, 156.5, 167.0, 169.1, 169.2, 170.5; ESIMS (*m*/*z*): $[M + Na]^+$ 1290 (100%), $[M + H]^+$ 1268 (40); HRMS–ESI *m*/*z*: calcd for $C_{70}H_{90}N_7O_{13}S$ 1268.6317, found 1268.6311.

Benzyl 4-((3*R*,6*R*)-3-guanidinopropyl-6-butylamino-9-((*S*)-2'-(3-methylbutoxy)-1,1'-binaphth-2-yloxy)-1,4,7-triaza-2,5,8-trioxononane)tetrahydro-2*H*-pyran-4-carboxylate dihydrochloride (2d)

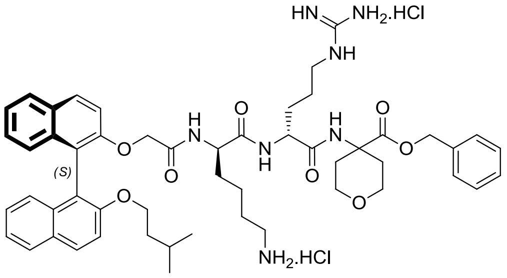

The Boc-protected **8d** (150 mg, 0.12 mmol) was added to a mixture of trifluoroacetic acid and CH_2Cl_2 3:7 (20 mL), and the solution was stirred at rt. After 2h, the solvent was evaporated under reduced pressure, HCl in ether (2 M solution, 3 mL) was added, and after a few minutes shaking, the solvent was evaporated under reduced pressure. This process was repeated two more times. The dihydrochloride salt **2d** (60 mg, 50%) was obtained by precipitation using anhydrous ether followed by filtration and vacuum drying. $[\alpha]^{24}_{D}$ −21.6 (*c* 0.2, MeOH); ^{1}H NMR (500 MHz, CD_3OD) δ 0.53 (d, J = 6.5 Hz, 3H), 0.58 (d, J = 6.5 Hz, 3H), 0.87–1.01 (m, 2H), 1.03–1.33 (m, 4H), 1.34–1.80 (m, 7H), 1.95–2.18 (m, 4H), 2.62–2.89 (m, 2H), 3.00–3.16 (m, 2H), 3.67–3.73 (m, 2H), 3.74–3.89 (m, 2H), 3.97–3.99 (m, 1H), 4.07–4.23 (m, 2H), 4.26-4.39 (m, 1H), 4.47 and 4.56 (ABq, J = 14.5 Hz, 2H), 5.10 and 5.14 (ABq, J = 12.5 Hz, 2H), 7.07 (dd, J_1 = 15.5 Hz, J_2 = 8.5 Hz, 2H), 7.22 (t, J = 8.0 Hz, 2H), 7.28–7.42 (m, 7H), 7.48 (d, J = 8.0 Hz, 1H), 7.56 (d, J = 9.0 Hz, 1H), 7.91 (d, J = 7.5 Hz, 2H), 8.02-8.04 (m, 2H); ^{13}C NMR (125 MHz, CD_3OD) δ 21.4, 21.7, 22.1, 24.5, 25.1, 26.8, 28.9, 31.3, 31.9, 32.6, 38.2, 39.6, 41.0, 52.3, 53.1, 56.8, 63.2, 63.5, 67.1, 68.0, 114.9, 115.9, 119.4, 120.7, 123.7, 124.1, 124.8, 125.2, 126.4, 126.5, 128.0, 128.2, 128.3, 128.5, 129.6, 129.8, 130.3, 133.9, 134.1, 136.1, 152.9, 154.8, 157.4, 171.9, 172.7, 173.1; ESIMS (*m*/*z*): $[M + H]^+$ 916 (40%), $[M + 2H]^{2+}$ 459 (100); HRMS–ESI *m*/*z*: calcd for $C_{52}H_{66}N_7O_8$ 916.4973, found 916.4982.

Benzyl 2-(4-(*tert*-butoxycarbonylamino) tetrahydro-2*H*-pyran-4-yl)acetate

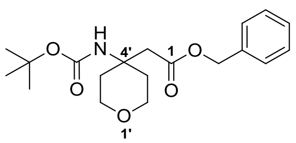

Benzyl bromide (0.25 mL, 2 mmol) was added to a stirred suspension of 2-(4-(*tert*-butoxycarbonylamino)tetrahydro-2*H*-pyran-4-yl)acetic acid (259 mg, 1.0 mmol) and anhydrous K_2CO_3 (345 mg, 2.5 mmol) in anhydrous THF (30 mL). The mixture was then heated under reflux for 3 h before being cooled, poured into water (50 mL) and extracted with EtOAc (3 × 50 mL). The combined organic layers were washed with sat. $NaHCO_3$ and sat. NaCl solution and dried (Na_2SO_4). After evaporation of the solvent, the residue was subjected to silica gel column chromatography with EtOAc/petroleum ether 0–20:100–80 to give the title compound (220 mg, 63%). ^{1}H NMR (500 MHz, $CDCl_3$) δ 1.38 (s, 9H), 1.61–1.71 (m, 2H), 1.99–2.09 (m, 2H), 2.80 (s, 2H), 3.62–3.70 (m, 4H), 4.42 (br s, 1H, NH), 5.16 (s, 2H), 7.21–7.32 (m, 5H); ^{13}C NMR (125 MHz, $CDCl_3$) δ 28.6, 35.7, 42.2, 51.3, 63.5, 66.4, 128.5, 128.6, 128.9, 136.1, 156.6, 170.7; ESIMS (*m*/*z*): $[M + H]^+$ 350 (30%).

Hydrochloride salt of benzyl 2-(4-aminotetrahydro-2*H*-pyran-4-yl)acetate (4e)

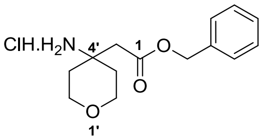

The above Boc-protected compound (220 mg, 0.63 mmol) was added to a mixture of trifluoroacetic acid and CH_2Cl_2 3:7 (20 mL), and the solution stirred at rt for 2 h. The solvent was then evaporated under the reduced pressure, and HCl in ether (2 M solution, 3 mL) was added. After a few minutes shaking, the solvent was evaporated under reduced pressure. This process was repeated two more times. The HCl salt of **4e** (180 mg, 100%) was obtained by precipitation using anhydrous ether. ^{1}H NMR (500 MHz, $CDCl_3$) δ 1.90–1.94 (m, 2H), 2.21–2.24 (m, 2H), 3.02 (s, 2H), 3.60–3.64 (m, 2H), 3.98–4.02 (m, 2H), 5.18 (s, 2H), 7.33–7.36 (m, 5H), 8.83, (br s, 3H, NH_3^+); ^{13}C NMR (125 MHz, $CDCl_3$) δ 33.9, 39.8, 53.7, 62.8, 67.5, 128.5, 128.7, 128.8, 134.9, 170.3; ESIMS (*m*/*z*): $[M + H]^+$ 250 (70%).

Benzyl 2-(4-(3-amino-6-(3-[(2,3-dihydro-2,2,4,6,7-pentamethyl-2*H*-1-benzofuran-5-yl)sulfonyl]guanidino)-1-aza-2-oxohexan)tetrahydro-2*H*-pyran-4-yl)acetate (6e)

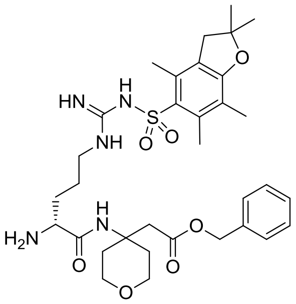

The title compound was prepared in two steps. The initial coupling reaction following protocol 1, by using **4e** (162 mg, 0.6 mmol) and Fmoc-(*R*)-Arg(Pbf)-OH (453 mg, 0.75 mmol) to yield the Fmoc protected precursor **5e** as a white foamy solid (300 mg, 57%); ESIMS (*m*/*z*): $[M + H]^+$ 881 (100%). Piperidine (0.5 mL), was added to a solution of **5e** (300 mg, 0.34 mmol) in CH_3CN (20 mL). The

mixture was stirred at rt for 3 h. After evaporation of the solvent under reduced pressure, the residue was subjected to flash silica gel column chromatography with MeOH/CH_2Cl_2 (0–5:100–95 to give **6e** as a white foam (165 mg, 78%). ^1H NMR (500 MHz, $CDCl_3$) δ 1.41 (s, 6H), 1.50–1.53 (m, 2H), 1.64–1.69 (m, 4H), 1.77–2.00 (m, 2H, NH_2), 2.04 (s, 3H), 2.14–2.18 (m, 2H), 2.43 (s, 3H), 2.53 (s, 3H), 2.84 (s, 2H), 2.90 (s, 2H), 3.12–3.13 (m, 2H), 3.20–3.30 (m, 1H), 3.50–3.54 (m, 2H), 3.65–3.67 (m, 2H), 5.01 (s, 2H), 6.41 (br s, 3H, 3NH), 7.24–7.28 (m, 5H), 7.45 (br s, 1H, NH); ^{13}C NMR (125 MHz, $CDCl_3$) δ 12.8, 18.2, 19.6, 25.9, 28.9, 32.1, 35.2, 35.3, 41.0, 42.6, 43.5, 52.0, 53.8, 55.1, 63.6, 66.5, 86.7, 117.8, 125.0, 128.6, 128.7, 128.9, 132.4, 133.2, 136.1, 138.5, 156.7, 159.0, 170.6, 175.6; ESIMS (*m*/*z*): $[M + H]^+$ 658 (100%).

Benzyl 2-(4-((3*R*,6*R*)-3-(3-[(2,3-dihydro-2,2,4,6,7-pentamethyl-2*H*-1-benzofuran-5-yl)sulfonyl]guanidino)propyl)-6-(4-(*tert*-butoxycarbonylamino)butyl)-9-((*S*)-2'-(3-methylbutoxy)-1,1'-binaphth-2-yloxy)-1,4,7-triaza-2,5,8-trioxononane)tetrahydro-2*H*-pyran-4-yl)acetate (8e)

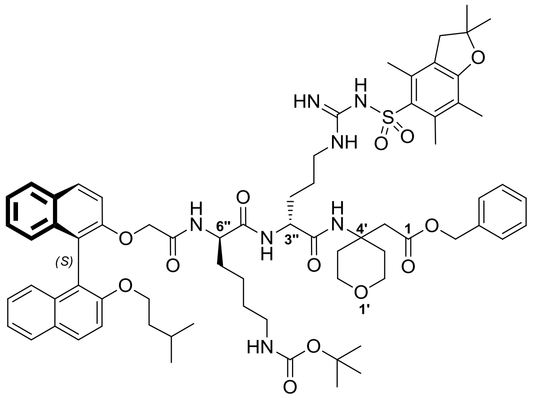

To a solution of **7** (160 mg, 0.25 mmol) and **8e** (150 mg, 0.23 mmol) in CH_3CN (20 mL), was added EDCI·HCl (100 mg, 0.50 mmol) and HOBt (54 mg, 0.50 mmol) and the resulting mixture was stirred at rt for 16 h. The reaction mixture was diluted with EtOAc (50 mL) and the EtOAc solution was washed with sat. aq. $NaHCO_3$ and water, dried ($MgSO_4$), and filtered. The solvent was removed by rotary evaporation and the residue subjected to flash column chromatography on silica gel with MeOH/CH_2Cl_2 0–1:100–99 as eluent to afford **8e** (235 mg, 85%). $[\alpha]^{24}_D$ −17.6 (*c* 0.3, MeOH); ^1H NMR (500 MHz, $CDCl_3$) δ 0.43 (d, *J* = 6.5 Hz, 3H), 0.48 (d, *J* = 6.5 Hz, 3H), 0.68–0.82 (m, 2H), 0.84–0.96 (m, 1H), 1.02–1.42 (m, 24H), 1.60–1.67 (m, 2H), 1.70–1.82 (m, 1H), 2.00 (s, 3H), 2.15–2.22 (m, 2H), 2.43 (s, 3H), 2.51 (s, 3H), 2.74–2.90 (m, 6H), 2.97–3.14 (m, 2H), 3.49–3.55 (m, 2H), 3.63–3.66 (m, 2H), 3.80–3.84 (m, 2H), 3.94–3.98 (m, 1H), 4.15–4.26 (m, 1H), 4.33 and 4.47 (ABq, *J* = 14.5 Hz, 2H), 4.58–4.69 (m, 1H), 4.98 (s, 2H), 6.01–6.02 (m, 3H, 3NH), 6.39 (br s, 1H, NH), 6.82 (br s, 1H, NH), 7.05–7.09 (m, 2H), 7.17–7.31 (m, 10H), 7.38 (d, *J* = 8.5 Hz, 1H), 7.79 (t, *J* = 8.5 Hz, 2H), 7.86–7.91 (m, 2H); ^{13}C NMR (125 MHz, $CDCl_3$) δ 12.5, 18.0, 19.4, 22.1, 22.4, 22.6, 24.5, 26.0, 28.5, 28.6, 28.6, 29.2, 31.3, 34.5, 34.7, 38.0, 40.2, 40.5, 42.6, 43.2, 52.5, 53.5, 63.2, 63.3, 66.2, 68.1,

78.9, 114.3, 115.7, 117.5, 119.3, 120.5, 123.9, 124.2, 124.7, 124.9, 125.6, 126.6, 126.7, 128.0, 128.1, 128.2, 128.5, 129.2, 129.8, 129.9, 132.2, 133.0, 133.6, 133.8, 135.9, 138.3, 152.2, 154.5, 156.0, 156.4, 158.7, 169.2, 170.3, 171.4, 171.7; ESIMS (*m*/*z*): $[M + Na]^+$ 1305 (50%), $[M + H]^+$ 1282 (10); HRMS–ESI *m*/*z*: $C_{71}H_{92}N_7O_{13}S$; calc. 1282.6474, found 1282.6482.

Benzyl 2-(4-((3*R*,6*R*)-3-guanidinopropyl-6-butylamino-9-((*S*)-2'-(3-methylbutoxy)-1,1'-binaphth-2-yloxy)-1,4,7-triaza-2,5,8-trioxononane)tetrahydro-2*H*-pyran-4-yl)acetate dihydrochloride (2e)

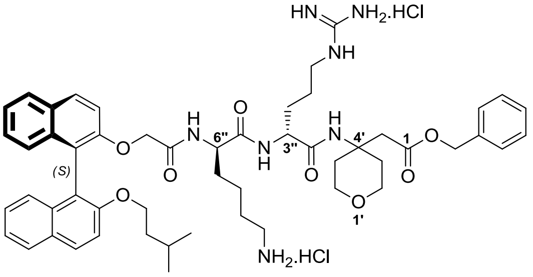

Boc-protected **8e** (200 mg, 0.156 mmol) was added to a mixture of trifluoroacetic acid and CH_2Cl_2 3:7 (20 mL). The solution was stirred at rt for 2 h, before the solvent was evaporated under reduced pressure. HCl in ether (2 M solution, 3 mL) was added and after a few minutes shaking, the solvent was evaporated under reduced pressure. This process was repeated two more times. The dihydrochloride salt **2e** (80 mg, 51%) was obtained by precipitation using anhydrous ether followed by filtration and vacuum drying. $[\alpha]^{24}_D$ −19.7 (*c* 0.2, MeOH); ^1H NMR (500 MHz, CD_3OD) δ 0.48 (d, *J* = 6.5 Hz, 3H), 0.53 (d, *J* = 6.5 Hz, 3H), 0.85–1.03 (m, 2H), 1.04–1.34 (m, 5H), 1.42–1.88 (m, 8H), 2.16–2.40 (m, 2H), 2.68–2.88 (m, 3H), 3.01–3.03 (m, 1H), 3.09 (br s, 2H), 3.53–3.84 (m, 4H), 3.90–4.04 (m, 1H), 4.08–4.33 (m, 3H), 4.47 and 4.57 (ABq, *J* = 14.5 Hz, 2H), 5.01–5.20 (m, 2H), 6.70 (br s, 1H, NH), 7.06 (t, *J* = 8.0 Hz, 2H), 7.16–7.44 (m, 9H), 7.48 (d, *J* = 8.0 Hz, 1H), 7.56 (d, *J* = 9.0 Hz, 1H), 7.91 (d, *J* = 7.5 Hz, 2H), 8.02 (d, *J* = 7.5 Hz, 2H), 8.12 (br s, 1H); ^{13}C NMR (125 MHz, CD_3OD) δ 21.4, 21.7, 22.1, 24.5, 25.1, 26.6, 28.8, 31.3, 34.5, 34.7, 38.2, 39.6, 40.9, 42.5, 52.3, 52.6, 53.9, 63.2, 63.3, 66.1, 67.9, 68.1, 114.8, 115.8, 119.4, 120.7, 123.7, 124.1, 124.8, 125.3, 126.4, 126.5, 128.0, 128.1, 128.5, 129.6, 129.7, 130.3, 134.0, 134.1, 136.4, 152.9, 154.8, 157.4, 170.5, 172.0, 172.9; MS (ESI, +ve) *m*/*z* 930 (40%) $[M + H]^+$; 466 (100) $[M + 2H]^{2+}$; HRMS–ESI *m*/*z*: calcd for $C_{53}H_{68}N_7O_8$ 930.5129, found 930.5133.

Benzyl 1-((*tert*-butoxycarbonylamino)methyl)cyclohexanecarboxylate

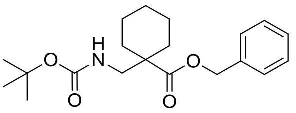

Benzyl bromide (0.25 mL, 2 mmol) was added to a stirred suspension of 1-((*tert*-butoxycarbonylamino)methyl)cyclohexanecarboxylic acid (257 mg, 1.0 mmol) and anhydrous K_2CO_3 (345 mg, 2.5 mmol) in anhydrous THF (30 mL). The mixture was heated under reflux for 3 h before being cooled,

poured into water (50 mL) and extracted with EtOAc (3 × 50 mL). The combined organic layers were washed with sat. $NaHCO_3$ and sat. NaCl solution, and dried (Na_2SO_4). After evaporation of the organic solvent, the residue was subjected to silica gel column chromatography with 0–20% EtOAc/petroleum ether 0–20:100–80 to give the benzyl ester (238 mg, 69%) as a colorless oil. ^{1}H NMR (500 MHz, $CDCl_3$) δ 1.30–1.65 (m, 8H), 1.41 (s, 9H), 1.92–2.01 (m, 2H), 3.31 (br s, 2H), 4.70 (br s, 1H, NH), 5.18 (s, 2H), 7.21–7.42 (m, 5H); ESIMS (*m*/*z*): $[M + H]^+$ 348 (30%).

Hydrochloride salt of benzyl 1-(aminomethyl)cyclohexanecarboxylate (4f)

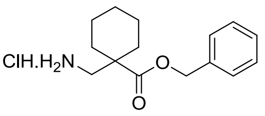

The above NBoc compound (230 mg, 0.69 mmol) was added to a mixture of trifluoroacetic acid and CH_2Cl_2 3:7 (20 mL), and the solution was stirred at rt for 2 h. The solvent was then evaporated under reduced pressure, HCl in ether (2 M solution, 3 mL) was added, and after a few minutes shaking, the solvent was evaporated under reduced pressure. This process was repeated two more times. The HCl salt of **4f** (200 mg, 100%) was obtained by precipitation using anhydrous ether. ^{1}H NMR (500 MHz, $CDCl_3$) δ 1.41–1.59 (m, 6H), 1.57–1.58 (m, 2H), 2.11–2.14 (m, 2H), 3.13 (s, 2H), 5.27 (s, 2H), 7.27–7.38 (m, 5H), 8.49 (br s, 3H, NH_3^+); ^{13}C NMR (125 MHz, $CDCl_3$) δ 22.3, 25.4, 31.6, 45.6, 45.9, 67.5, 128.4, 128.5, 128.8, 136.1, 174.4; ESIMS (*m*/*z*): $[M + H]^+$ 248 (70%).

Benzyl 1-((4*R*)-2-aza-7-((2,3-dihydro-2,2,4,6,7-pentamethyl-2*H*-1-benzofuran-5-yl)sulfonylguanidino))-4-(9*H*-9-fluorenylmethoxycarboxamido)-3-oxoheptan)cyclohexanecarboxylate (5f)

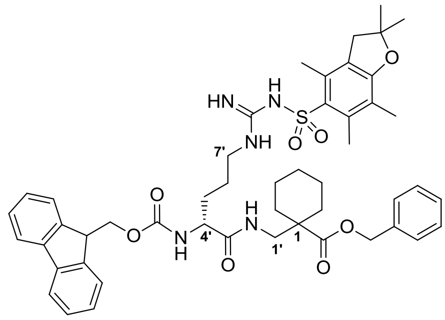

To a solution of Fmoc-(*R*)-Arg(Pbf)-OH (453 mg, 0.75 mmol) and **4f** (200 mg, 0.7 mmol) in CH_3CN (20 mL), was added EDCI·HCl (200 mg, 1.0 mmol), HOBt (108 mg, 0.8 mmol), and DIPEA (0.35 mL, 0.2 mmol) and the resulting mixture was stirred at rt for 16 h. The reaction was diluted with EtOAc (50 mL) and the EtOAc solution was washed with sat. aq. $NaHCO_3$ and water, dried ($MgSO_4$), and filtered. The solvent was removed by rotary evaporation, and the residue was then subjected to flash silica gel chromatography with MeOH in CH_2Cl_2 MeOH/CH_2Cl_2 (0–3:100–97) as eluent to afford **5f** (490 mg, 75%). ^{1}H NMR (500 MHz, $CDCl_3$) δ 1.10–1.19 (m, 6H), 1.44 (s, 6H), 1.24–1.32 (m, 5H), 1.36–1.44 (m, 1H), 1.98–2.05 (m, 2H), 2.09 (s, 3H), 2.53 (s, 3H), 2.61 (s,

3H), 2.93 (s, 2H), 3.22–3.24 (m, 3H), 3.25–3.31 (m, 1H), 4.16–4.19 (m, 2H), 4.36–4.37 (m, 2H), 5.07 and 5.11 (ABq, J = 12.5 Hz, 2H), 5.68 (br s, 1H, NH), 5.81 (br s, 1H, NH), 6.14 (br s, 2H, 2NH), 6.70 (br s, 1H, NH), 7.27–7.32 (m, 7H), 7.37–7.40 (m, 2H), 7.52–7.62 (m, 2H), 7.75 (d, J = 7.5 Hz, 2H); ^{13}C NMR (125 MHz, $CDCl_3$) δ 12.7, 18.2, 19.6, 22.9, 25.9, 28.9, 32.0, 32.4, 40.9, 43.5, 46.5, 48.1, 54.6, 66.9, 86.6, 117.7, 124.9, 128.4, 128.5, 128.8, 132.4, 133.3, 136.3, 138.5, 156.7, 158.9, 175.6, 175.7; ESIMS (*m*/*z*): $[M + Na]^+$ 900 (100%).

Benzyl 1-((4*R*)-2-aza-7-((2,3-dihydro-2,2,4,6,7-pentamethyl-2*H*-1-benzofuran-5-yl)sulfonylguanidino))-4-(9*H*-9-fluorenylmethoxycarboxamido)-3-oxoheptan)cyclohexanecarboxylate (6f)

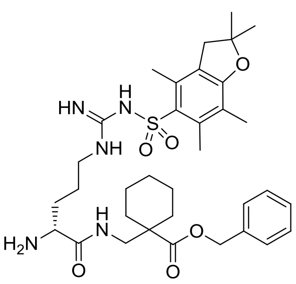

Piperidine (0.5 mL, 5.06 mmol) was added to a solution of **5f** (380 mg, 0.34 mmol) in CH_3CN (20 mL) and the mixture was stirred at rt for 3 h. After evaporation of solvent under reduced pressure, the residue was subjected to flash silica gel column chromatography with MeOH/CH_2Cl_2 (0–5:100–95) to give **6f** as a white foam (184 mg, 83%). ^{1}H NMR (500 MHz, $CDCl_3$) δ 1.21–1.30 (m, 5H), 1.35–1.74 (m, 9H/NH_2), 1.42 (s, 6H), 1.99–2.01 (m, 2H), 2.05 (s, 3H), 2.47 (s, 3H), 2.54 (s, 3H), 2.91 (s, 2H), 3.12–3.14 (m, 2H), 3.21–3.37 (m, 3H), 5.06 (s, 2H), 6.40 (br s, 3H, 3NH), 7.24–7.30 (m, 5H), 7.56 (br s, 1H, NH); ^{13}C NMR (125 MHz, $CDCl_3$) δ 12.7, 18.2, 19.6, 25.7, 28.8, 29.5, 31.9, 32.4, 32.9, 40.8, 43.5, 53.7, 54.4, 56.6, 63.6, 63.7, 67.4, 86.7, 117.8, 124.9, 128.4, 128.6, 128.8, 132.4, 133.1, 135.8, 138.5, 156.6, 159.0, 173.3, 175.5; ESIMS (*m*/*z*): $[M + H]^+$ 644 (100%).

Benzyl 1-((4*R*,7*R*)-4-(3-[(2,3-dihydro-2,2,4,6,7-pentamethyl-2*H*-1-benzofuran-5-yl)sulfonyl]guanidinopropyl)-10-((*S*)-2'-(3-methylbutoxy)-1,1'-binaphth-2-yloxy)-7-(4-(*tert*-butoxycarbonylamino)butyl)-2,5,8-triaza-3,6,9-trioxodecan)cyclohexanecarboxylate (8f)

To a solution of **7** (192 mg, 0.30 mmol) and **6f** (150 mg, 0.23 mmol) in CH_3CN (20 mL), was added EDCI·HCl (100 mg, 0.50 mmol) and HOBt (54 mg, 0.50 mmol), and the resulting mixture was stirred at rt for 16 h. The reaction was diluted with EtOAc (50 mL) and the EtOAc solution was washed with sat. aq. $NaHCO_3$ and water, dried ($MgSO_4$), and filtered. The solvent was removed by rotary evaporation and the residue was subjected to flash chromatography on silica gel with MeOH/CH_2Cl_2 0–1:100–99 as eluent to afford **8f** (244 mg, 83%). $[\alpha]^{24}_D$ −18.3 (*c* 0.5, MeOH); ^{1}H NMR (500 MHz, $CDCl_3$) δ 0.49 (d, J = 7.0 Hz, 3H),

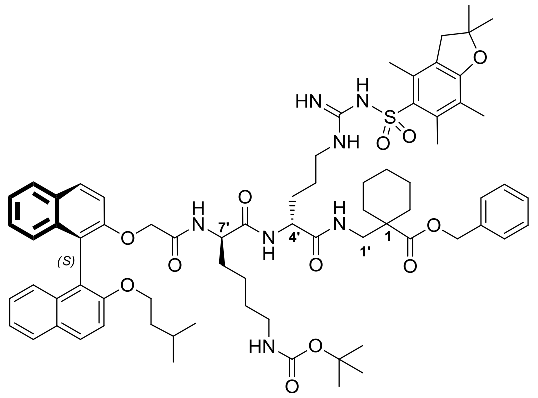

0.53 (d, J = 7.0 Hz, 3H), 0.80–0.81 (m, 2H), 0.89–0.95 (m, 2H), 1.09–1.61 (m, 16H), 1.42 (s, 9H), 1.428 (s, 3H), 1.43 (s, 3H), 1.69–1.80 (m, 1H), 1.94–2.05 (m, 2H), 2.07 (s, 3H), 2.50 (s, 3H), 2.57 (s, 3H), 2.86–3.20 (m, 4H), 2.92 (s, 2H), 3.27 (dd, J_1 = 13.5 Hz, J_2 = 6.0 Hz, 1H), 3.48 (dd, J_1 = 13.0 Hz, J_2 = 6.5 Hz, 1H), 3.86–3.90 (m, 1H), 3.90–4.00 (m, 1H), 4.01–4.04 (m, 1H), 4.22–4.32 (m, 1H), 4.41 and 4.50 (ABq, J = 14.5 Hz, 2H), 4.63–4.72 (m, 1H, NH), 5.08 (s, 2H), 6.10–6.11 (m, 3H, 2NH), 6.55 (br s, 1H, NH), 6.82 (br d, J = 7.5 Hz, 1H, NH), 7.13 (dd, J_1 = 12.5 Hz, J_2 = 8.5 Hz, 2H), 7.20–7.37 (m, 10H), 7.44 (d, J = 9.0 Hz, 1H), 7.86 (t, J = 8.5 Hz, 2H), 7.94 (d, J = 9.0 Hz, 2H); ^{13}C NMR (125 MHz, $CDCl_3$) δ 13.7, 19.2, 20.5, 23.3, 23.5, 23.7, 23.9, 23.9, 25.7, 26.8, 29.7, 29.8, 30.2, 30.9, 32.5, 32.8, 32.9, 39.1, 41.2, 41.5, 44.4, 47.7, 49.2, 53.7, 67.7, 69.2, 69.3, 80.0, 87.5, 115.5, 117.0, 118.6, 120.4, 121.7, 124.9, 125.3, 125.7, 126.1, 126.7, 127.7, 127.8, 129.1, 129.2, 129.3, 129.7, 130.3, 130.9, 131.0, 133.3, 134.2, 134.8, 135.0, 137.5, 139.4, 153.4, 155.6, 157.2, 157.5, 159.8, 170.2, 172.5, 172.9, 176.5; ESIMS (*m/z*): $[M + H]^+$ 1280 (40%); HRMS–ESI *m/z*: calcd for $C_{72}H_{94}N_7O_{12}S$ 1280.6681, found 1280.6675.

Benzyl 1-((4*R*,7*R*)-7-butylamino-4-(3-guanidinopropyl)-10-((*S*)-2'-(3-methylbutoxy)-1,1'-binaphth-2-yloxy)-2,5,8-triaza-3,6,9-trioxodecan)cyclohexanecarboxylate dihydrochloride (2f)

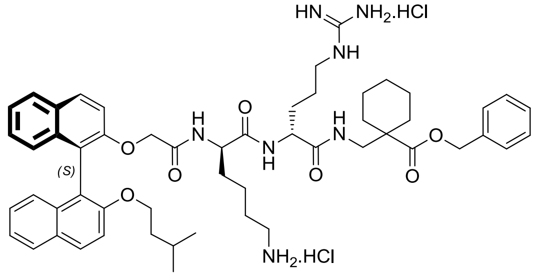

Compound **8f** (200 mg, 0.16 mmol) was added to a mixture of trifluoroacetic acid and CH_2Cl_2 (3:7, 20 mL) and the solution was stirred at rt for 2 h. The solvent was then evaporated under the reduced pressure, HCl in ether (2 M solution, 3 mL) was added, and after a few minutes shaking, the solvent was evaporated under reduced pressure. This process was repeated two more times. The salt **2f** (110 mg, 70%) was obtained by precipitation using anhydrous ether followed filtration and vacuum drying. $[\alpha]^{24}_{D}$ −20.1 (*c* 0.2, MeOH); ^{1}H NMR (500 MHz, CD_3OD) δ 0.51 (d, J = 6.5 Hz, 3H), 0.56 (d, J = 6.5 Hz, 3H), 0.85–1.05 (m, 2H), 1.13–1.78 (m, 19H), 2.00–2.17 (m, 2H), 2.70–2.91 (m, 2H), 3.07–3.21 (m, 2H), 3.21–3.24 (m, 1H), 3.46–3.49 (m, 1H), 3.95–3.98 (m, 1H), 4.11–4.15 (m, 2H), 4.27 (br s, 1H), 4.46 and 4.55 (ABq, J = 14.5 Hz, 2H), 5.11 (s, 2H), 7.04–7.08 (m, 2H), 7.20–

7.23 (m, 2H), 7.31–7.40 (m, 7H), 7.45 (d, *J* = 9.0 Hz, 1H), 7.55 (d, *J* = 8.5 Hz, 1H), 7.89–7.95 (m, 3H/NH), 8.02 (d, *J* = 9.0 Hz, 2H); ^{13}C NMR (125 MHz, CD_3OD) δ 21.3, 21.5, 22.0, 22.7, 24.3, 24.7, 25.1, 25.4, 26.5, 29.0, 31.0, 31.6, 31.7, 38.0, 39.1, 40.7, 52.3, 53.1, 66.5, 67.8, 68.0, 114.7, 115.7, 119.7, 120.4, 120.5, 123.5, 123.9, 124.7, 125.1, 126.2, 126.3, 127.8, 128.0, 128.3, 129.4, 129.6, 130.1, 132.3, 133.8, 134.0, 136.4, 143.3, 152.8, 154.7, 157.3, 171.9, 172.7, 175.1; ESIMS (*m*/*z*): $[M + H]^+$ 928 (40%), $[M + 2H]^{2+}$ 465 (100); HRMS–ESI *m*/*z*: calcd for $C_{54}H_{70}N_7O_7$ 928.5337, found 928.5343.

Benzyl 2-(*tert*-butoxycarbonylamino)-2-ethylbutanoate

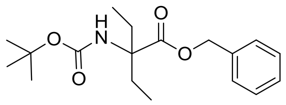

To 2-(*tert*-butoxycarbonylamino)-2-ethylbutanoic acid (165 mg, 0.71 mmol) and K_2CO_3 (400 mg, 2.87 mmol) in acetone (25 mL) was added benzyl bromide (0.2 mL, 1.68 mmol). The mixture was heated under reflux overnight before being cooled, filtered and evaporated to dryness. The resulting residue was dried overnight under vacuum to yield the protected acid (182 mg, 80%) as a colorless oil. ^{1}H NMR (300 MHz, $CDCl_3$) δ 0.71 (d, *J* = 7.5 Hz, 3H), 0.75 (d, *J* = 7.5 Hz, 3H), 1.42 (s, 9H), 1.74–1.86 (m, 2H), 2.16–2.56 (m, 2H), 5.17 (s, 2H), 5.50 (br s, 1H, NH), 7.29–7.36 (m, 5H); ^{13}C NMR(75 MHz, $CDCl_3$) δ 8.1, 28.2, 33.3, 64.6, 67.0, 78.8, 128.2, 128.3, 128.4, 135.3, 153.7, 173.7.

Benzyl 2-amino-2-ethylbutanoate (4g)

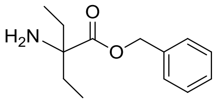

This compound was prepared following protocol 3 and basification, by using the above protected amino acid (180 mg, 0.56 mmol) to yield **4g** (116 mg, 94%) as a light brown oil. ^{1}H NMR (300 MHz, $CDCl_3$) δ 0.83 (d, *J* = 7.5 Hz, 3H), 0.85 (d, *J* = 7.5 Hz, 3H), 1.55–1.67 (m, 2H), 1.76–1.89 (m, 2H), 2.47 (br s, 2H, NH_2), 5.15 (s, 2H), 7.32–7.40 (m, 5H); ^{13}C NMR (75 MHz, $CDCl_3$) δ 8.1, 32.1, 62.1, 66.7, 128.1, 128.2, 128.4, 135.8, 176.4; ESIMS (*m*/*z*): $[M + H]^+$ 222 (100%).

Benzyl (5*R*)-3-aza-2,2-diethyl-5-(9*H*-9-fluorenylmethoxycarboxamido)-8-(3-[(2,3-dihydro-2,2,4,6,7-pentamethyl-2*H*-1-benzofuran-5-yl)sulfonyl]guanidino)-4-oxooctanoate (5g)

To a solution of Fmoc-(*R*)-Arg(Pbf)-OH (600 mg, 0.92 mmol) and **4g** (210 mg, 0.95 mmol) in acetonitrile (25 mL) was added DIPEA (0.4 mL), followed by BOP reagent (600 mg, 1.36 mmol). The reaction mixture was stirred at rt for 3 h before the solvent was evaporated. The residue was subjected to column flash chromatography (MeOH/CH_2Cl_2 (5:95) to yield **5g**

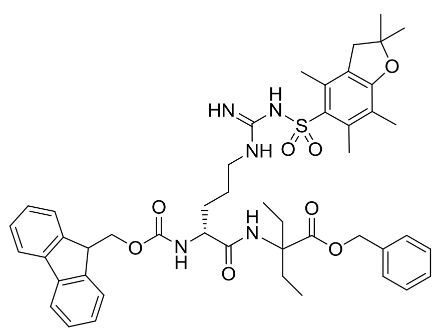

(402 mg, 50%) as an off-white foam. ^{1}H NMR (300 MHz, $CDCl_3$) δ 0.63–0.68 (m, 6H), 1.41 (s, 6H), 1.50–1.70 (m, 3H), 1.75–1.89 (m, 3H), 2.06 (s, 6H), 2.11–2.24 (m, 2H), 2.50 (s, 3H), 2.57 (s, 3H), 2.89 (s, 2H), 3.12–3.27 (m, 2H), 4.11–4.15 (m, 1H), 4.15–4.24 (m, 1H), 4.26–4.34 (m, 1H), 5.13 (s, 2H), 5.97 (d, *J* = 7.5 Hz, 1H, NH), 6.08 (br s, 1H, NH), 6.23 (s, 2H, 2NH), 7.12 (br s, 1H, NH), 7.20–7.38 (m, 9H), 7.54 (d, *J* = 7.5 Hz, 2H), 7.72 (d, *J* = 7.5 Hz, 2H); ESIMS (*m*/*z*): $[M + H]^+$ 852 (100%).

Benzyl (5*R*)-5-amino-3-aza-2,2-diethyl-8-(3-[(2,3-dihydro-2,2,4,6,7-pentamethyl-2*H*-1-benzofuran-5-yl)sulfonyl]guanidino)-4-oxooctanoate (6g)

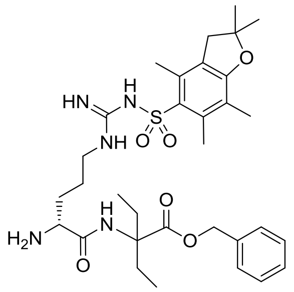

This compound was prepared following protocol 2, by using **5g** (402 mg, 0.47 mmol) to yield **6g** (187 mg, 63%). ^{1}H NMR (500 MHz, $CDCl_3$) δ 0.66–0.71 (m, 6H), 1.33 (s, 3H), 1.34 (s, 3H), 1.45 (s, 9H), 1.57–1.67 (m, 3H), 1.80–1.89 (m, 3H), 2.08 (s, 3H), 2.13–2.19 (m, 2H), 2.49 (s, 3H), 2.56 (s, 3H), 2.94 (s, 2H), 3.05–3.25 (m, 2H), 3.57–3.66 (m, 1H), 5.15 (s, 2H), 6.42 (s, 2H, 2NH), 6.52 (br s, 1H, NH), 7.29–7.35 (m, 5H), 7.96 (s, 1H, NH); ESIMS (*m*/*z*): $[M + H]^+$ 630 (100%).

Benzyl (5*R*,8*R*)-2,2-diethyl-5-(3-[(2,3-dihydro-2,2,4,6,7-pentamethyl-2*H*-1-benzofuran-5-yl)sulfonyl]guanidinopropyl)-11-((*S*)-2'-(3-methylbutoxy)-1,1'-binaphth-2-yloxy)-8-(4-(*tert*-butoxycarbonylamino)butyl)-3,6,9-triaza 4,7,10-trioxoundecanoate (8g)

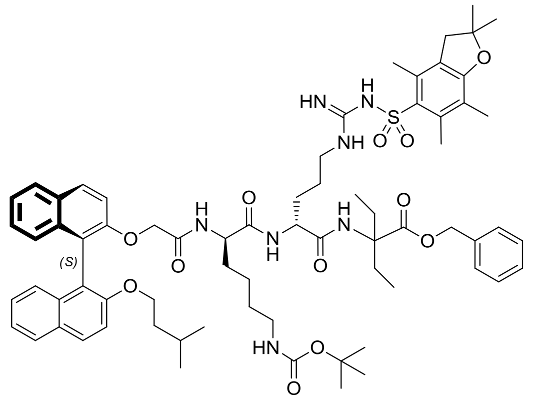

To a solution of **7** (50 mg, 0.078 mmol) and **6g** (48 mg, 0.076 mmol) in acetonitrile (10 mL) was added HOBt (30 mg, 0.222 mmol), followed by EDCI (30 mg, 0.156 mmol). The mixture was stirred at rt for 3 days before the solvent was removed. The residue was subjected to radial chromatography MeOH/CH_2Cl_2 1–2:99–98 to yield **8g** (54 mg, 55%) as an off-white solid. ^{1}H NMR (500 Hz, $CDCl_3$) δ 0.50 (d, *J* = 7.0 Hz, 3H), 0.54 (d, *J* = 6.5 Hz, 3H), 0.64–0.71 (m, 6H), 0.80–0.89 (m, 3H), 0.94–0.96 (m, 1H), 1.11–1.59 (m, 12H), 1.33 (s, 3H), 1.34 (s, 3H), 1.41 (s, 9H), 1.79–1.87 (m, 3H), 2.07 (s, 3H), 2.26–

2.35 (m, 2H), 2.49 (s, 3H), 2.55 (s, 3H), 2.90–2.97 (m, 2H), 2.92 (s, 3H), 3.08–3.20 (m, 2H), 3.88–3.93 (m, 1H), 4.05–4.09 (m, 2H), 4.25–4.32 (m, 1H), 4.41 and 4.49 (ABq, *J* = 12.0 Hz, 2H), 4.74–4.76 (m, 1H, NH), 5.19 (s, 2H), 6.18–6.20 (m, 3H, 3NH), 7.12 (d, *J* = 8.0 Hz, 1H), 7.16 (d, *J* = 7.5 Hz, 1H), 7.20–7.37 (m, 10H), 7.44 (d, *J* = 9.0 Hz, 1H), 7.83 (d, *J* = 7.5 Hz, 1H), 7.87 (d, *J* = 8.0 Hz, 1H), 7.92 (d, *J* = 9.0 Hz, 1H), 7.94 (d, *J* = 8.5 Hz, 1H); ^{13}C NMR (126 MHz, $CDCl_3$) δ 8.3, 12.4, 17.9, 19.3, 21.6, 22.1, 22.3, 22.6, 24.5, 25.6, 27.4, 27.5, 28.4, 28.5, 29.1, 31.0, 37.9, 40.0, 40.4, 43.2, 52.7, 53.5, 65.6, 67.4, 68.0, 68.2, 78.9, 86.2, 114.2, 115.8, 117.3, 119.3, 120.4, 123.8, 124.1, 124.5, 124.9, 125.5, 126.6, 127.9, 128.0, 128.2, 128.5, 128.6, 129.1, 129.6, 129.8, 132.2, 133.1, 133.6, 133.8, 135.3, 138.3, 152.2, 154.4, 156.2, 158.6, 169.1, 170.2, 171.5, 173.6; ESIMS (*m*/*z*): $[M + H]^+$ 1254 (100%).

Benzyl (5*R*,8*R*)-2,2'-diethyl-8-butylamino-5-(3-guanidinopropyl)-11-((*S*)-2'-(3-methylbutoxy)-1,1'-binaphth-2-yloxy)-3,6,9-triaza-4,7,10-trioxoundecanoate dihydrochloride (2g)

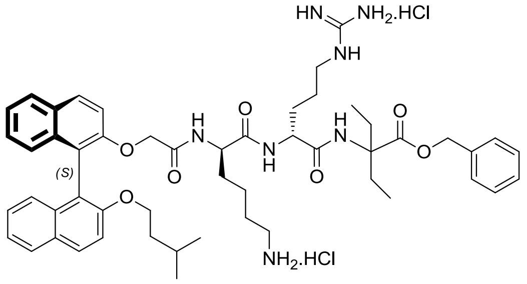

This compound was prepared following protocol 3, by using **8g** (54 mg, 0.043 mmol) to yield **2g** (37 mg, 88%) as an off-white solid. ^{1}H NMR (300 MHz, CD_3OD) δ 0.53 (d, *J* = 6.1 Hz, 3H), 0.58 (d, *J* = 6.3 Hz, 3H), 0.73–0.80 (m, 6H), 0.91–0.98 (m, 2H), 1.08–1.30 (m, 5H), 1.49–1.69 (m, 7H), 1.77–2.19 (m, 5H), 2.74–2.80 (m, 2H), 3.10–3.15 (m, 2H), 3.93–4.01 (m, 1H), 4.13–4.22 (m 2H), 4.26–4.30 (m, 1H), 4.45 and 4.56 (ABq, *J* = 14.7, 2H), 5.18 (s, 2H), 7.07 (dd, J_1 = 8.4 Hz, J_2 = 4.5 Hz, 2H), 7.19–7.25 (m, 2H), 7.31–7.40 (m, 7H), 7.48 (d, *J* = 9.0 Hz, 1H), 7.55 (d, *J* = 9.3 Hz, 1H), 7.91 (d, *J* = 8.1 Hz, 2H), 8.01 (dd, J_1 = 9.0 Hz, J_2 = 7.2 Hz, 2H); ^{13}C NMR (75 MHz, CD_3OD) δ 8.3, 8.4, 22.6, 22.8, 23.2, 25.6, 26.3, 27.4, 27.8, 28.0, 30.0, 32.5, 39.3, 40.4, 41.9, 53.3, 54.4, 65.6, 68.3, 69.0, 69.2, 116.0, 116.9, 120.5, 124.8, 125.2, 126.0, 126.4, 127.5, 127.6, 129.1, 129.3, 129.49, 129.5, 129.6, 130.7, 130.8, 130.9, 131.4, 135.1, 135.2, 137.1, 154.0, 155.9, 158.5, 170.7, 172.7, 173.1, 174.6; ESIMS (*m*/*z*): $[M + H]^+$ 902 (15%); $[M + 2H]^{2+}$ 452 (100); HRMS–ESI *m*/*z*: calcd for $C_{52}H_{68}N_7O_7$ 902.5180, found 902.5220.

References

1. Bremner, J. B.; Keller, P. A.; Pyne, S. G.; Boyle, T. P.; Brkic, Z.; David, D. M.; Morgan, J.; Robertson, M.; Somphol, K.; Miller, M. H.; Howe, A. S.; Ambrose, P.; Bhavnani, S.; Fritsche, T. R. Biedenbach, D. J.; Jones, R. N.; Buckheit, R. W., Jr.; Watson, K. M.; Baylis, D.; Coates, J. A.; Deadman, J.; Jeevarajah, D.; McCracken, A.; Rhodes, D. I. *Angew. Chem., Int. Ed*. **2010**, *49*, 537–540. doi:10.1002/anie.200904392
2. Tailleur, P.; Berlinguet, L. *Can. J. Chem.* **1961,** *39*, 1309–1320. doi:10.1139/v61-165
